# Supplementary material for: Professionals’ perspectives on factors affecting GP trainees’ patient mix: results from an interview and focus group study among professionals working in Dutch general practice
Source: BMJ Open. 2019 Dec 15;9(12):e032182. doi: 10.1136/bmjopen-2019-032182 (PMC6924856; doi:10.1136/bmjopen-2019-032182)
Supplement: Supplementary data [file bmjopen-2019-032182supp001.pdf]

Appendix I

Theme	Subtheme	Summary
Disease characteristics & patient – doctor relationship		Patients can have a strong preference for which doctor they want to consult. This preference is dependent on their complaint. Patients prefer to consult a trusted doctor when they have a complex complaint, and for minor ailments patients have less preference.
Overcoming lack in patient – doctor relationship		The time that trainees spend in a practice is relatively short for establishing a trusted relationship with patients. However, this lack in bond between trainee and patient can be surmounted.
	Transference of trust	Team members, who are trusted by patients, can 'transfer' this trust to the trainee. By doing so, they enhance bonding between trainee and patient.
	Acquisition of trust	Through good initial contact between trainees and patients, trainees can form a bond with patients.
Influencing factors		Whether or not a bond forms between trainees and patients depend on three factors
	Trainees behaviour	Trainees can directly and indirectly influence their patient mix. They can do this directly through bonding with patient by being an understanding, empathic doctor. Indirectly, trainees can influence their patient mix by influencing the willingness and trust team members have in them.
	Attitudes of team members	Team members can influence trainees' patient mix deliberately. However, whether or not they are willing to do so, depends on the relationship they have with the trainee, the trust they have in trainees' capabilities and the involvement in the education of the trainee.
	Context	The context of a training practice can hinder or help trainees' patient mix. It can also affects the strength in bond between doctors and patients.

Table 5 - Summary of the major themes and their subthemes
