# Supplementary material for: Professionals’ perspectives on factors affecting GP trainees’ patient mix: results from an interview and focus group study among professionals working in Dutch general practice
Source: BMJ Open. 2019 Dec 15;9(12):e032182. doi: 10.1136/bmjopen-2019-032182 (PMC6924856; doi:10.1136/bmjopen-2019-032182)
Supplement: Author's manuscript [file bmjopen-2019-032182.draft_revisions.pdf]

BMJ Open is committed to open peer review. As part of this commitment we make the peer review history of every article we publish publicly available.

When an article is published we post the peer reviewers' comments and the authors' responses online. We also post the versions of the paper that were used during peer review. These are the versions that the peer review comments apply to.

The versions of the paper that follow are the versions that were submitted during the peer review process. They are not the versions of record or the final published versions. They should not be cited or distributed as the published version of this manuscript.

BMJ Open is an open access journal and the full, final, typeset and author-corrected version of record of the manuscript is available on our site with no access controls, subscription charges or pay-per-view fees (http://bmjopen.bmj.com).

If you have any questions on BMJ Open's open peer review process please email info.bmjopen@bmj.com

BMJ Open

What shapes trainees' patient mix? A qualitative study among professionals in general practice

Journal:	*BMJ Open*
Manuscript ID	bmjopen-2019-032182
Article Type:	Research
Date Submitted by the Author:	06-Jun-2019
Complete List of Authors:	Bever, Sarah; Amsterdam UMC - Locatie AMC, Department of General Practice/ GP Specialty Training Program<br>van Rhijn, Suzanne; Amsterdam UMC - Locatie AMC, Department of General Practice/ GP Specialty Training Program<br>van Dijk, Nynke; Amsterdam UMC - Locatie AMC<br>Kramer, Anneke ; Leiden University Medical Center, Department of Public Health and Primary Care<br>Visser, Mechteld; Amsterdam UMC - Locatie AMC
Keywords:	MEDICAL EDUCATION & TRAINING, PRIMARY CARE, QUALITATIVE RESEARCH

SCHOLARONE™
Manuscripts

What shapes trainees' patient mix? A qualitative study among professionals in general practice

Authors: Sarah de Bever (MD)[a] , Suzanne C. van Rhijn (BSc)[a], Nynke van Dijk (MD, PhD)[a], Anneke W.M. Kramer (MD, PhD)[b], Mechteld R.M. Visser (PhD)[a]

Affliations:
a : Amsterdam UMC, University of Amsterdam, Department of General Practice/ GP Specialty Training Program, Amsterdam Public Health Research Institute, Amsterdam, Netherlands
b: Department of Public Health and Primary Care, Leiden University Medical Centre, Leiden, the Netherlands

Corresponding author: s.debever@amsterdamumc.nl. Adress: Meibergdreef 15, room J2-113, 1105 AZ Amsterdam, the Netherlands

Contact information:
Suzanne C. van Rhijn: suzannevanrhijn@hotmail.com
Nynke van Dijk: n.vandijk@amc.uva.nl
Anneke W.M. Kramer: a.w.m.kramer@lumc.nl
Mechteld. R.M. Visser: m.r.visser@amc.uva.nl

For peer review only

Word count: 3813

Abstract

Introduction: Seeing and treating patients in daily practice forms the basis of general practitioner (GP) training. Yet the mix of patients seen by GP trainees does not always match their educational needs. Knowledge about the factors that shape this mix is limited, especially with regard to the role of the professionals who work in the GP practice.

Aim: We investigated factors affecting the mix of patients seen by GP trainees from the professionals' perspective.

Design & Setting: This qualitative study involved GP trainees, GP supervisors, medical receptionists, and nurse practitioners affiliated with a GP Specialty Training Institute in the Netherlands.

Methods : Twelve focus groups and seven interviews with 73 participants were held. Data collection and analysis were iterative using thematic analysis with a constant comparison methodology.

Results: The characteristics of patients' health problem and the bond between doctor and patient are important determinants of GP trainees' patient mix. Trainees have not yet developed a bond with patients As a consequence they are less likely to see patients with complex health problems. However, trainees can deliberately influence their patient mix by paying purposeful attention to bonding with patients and by gaining professional trust through focused engagement with practice colleagues.

Conclusion: Trainees' patient mix is affected by various factors. Trainees and team members can take steps to overcome this problem, but whether this happens depend on the interaction between trainees' behaviour, the attitudes of team members, and the context. The findings show how the mix of patients seen by trainees can be influenced to become more trainee centred and learning oriented.

For peer review only - http://bmjopen.bmj.com/site/about/guidelines.xhtml

Keywords: *Postgraduate Medical Education, General Practice, Doctor-Patient Relationship. Workplace-Based Learning, Trust*

For peer review only

Article summary – strengths & limitations of the study

- We conducted focus groups and interviewed with 73 professionals in total, who together represented all professional roles in GP practice (GP, medical receptionist, nurse practitioner, GP trainee), providing a broad point of view on trainees' patient mix.
- Qualitative analysis allowed for an in-depth exploration on factors affecting trainees' patient mix
- Using professionals this study offers a novel perspective on trainees patient mix
- Perspectives of patients' are missing, though literature on their perspectives suggest similar findings.
- The result can be used in settings were continuity of care is of importance, however in other settings this may be less likely.

For peer review only - http://bmjopen.bmj.com/site/about/guidelines.xhtml

Introduction

Seeing and treating patients forms the basis of training for medical graduates specializing to become general practitioners (GPs)(1, 2). Thus their development of competence and skills depends on the mix of patients seen. According to Ericsson's theory of deliberate practice, mastery of competencies is achieved through repetitive practice, monitoring, and timely feedback from a trusted coach followed by further practice(3, 4). In the clinical workplace setting, this means repetitive cycles of the same patient symptoms or situations. Yet in everyday practice the mix of patients seen by GP trainees cannot always be organized in this way(5).The mix seems to be affected by a number of factors other than the educational needs of the trainee(6, 7). To make GP training more effective, a thorough understanding of all factors affecting the patient mix of GP trainees is needed.

Previous studies of trainees' patient mix mostly focused on patients' willingness to consult the trainee(8-11). However, trainees, their supervisors, and the practice environment probably also affect the patient mix. It has been found, for example, that GP trainees influence their patient mix in order to fill their knowledge gaps(12), but this requires developmental space(2, 13). Supervisors can provide this space, however they need to trust the trainee first(14-16). The relationship between GP trainee and supervisor affects the trainee's ability to steer his/her learning, and therefore probably also their patient mix(2, 12). Contextual factors, such as the work climate, may also influence which patients are seen by the trainee. While trainee workplaces should 'invite and support' learning(17-19), some are more work-oriented than supportive for learners(6, 20). For example, medical receptionists are responsible for allocating patients and they may not necessarily prioritize a trainee's educational needs when allocating patients. Many studies have investigated how trainees learn(1, 12, 21, 22), how supervisors develop their trust(23, 24), the importance of work relationships, and the

For peer review only - http://bmjopen.bmj.com/site/about/guidelines.xhtml

influence of context on learning(2, 12, 19), but none have looked at the impact of the trainee's patient mix. Yet this information is needed in order to support training in the general practice. In this study, we investigated factors influencing the patient mix of GP trainees. To this end, we carried out a qualitative study involving different health professionals (GP trainees, their supervisors, medical receptionists, and nurse practitioners) in a general practice setting. Findings can be used to ensure that trainees see a mix of patients appropriate to their needs, thereby optimizing workplace-based learning.

For peer review only

For peer review only - http://bmjopen.bmj.com/site/about/guidelines.xhtml

Methods

Design

Focus group meetings and additional semi-structured interviews were used in this qualitative study. Focus groups were chosen because they provide the opportunity for in-depth investigation of a topic through group discussion(25). After the initial analysis, we held six interviews with trainees to deepen our first understandings. We purposively sampled on third year trainees, because they appeared to be the most informative participants.

Setting

In the Netherlands all residents are registered with a general practice. GPs are generalists, gatekeepers for specialist and hospital care, and patients' first contact with the healthcare system (26). Most general practices are in the community, so that there is often a strong, long-standing relationship between GPs and patients(26). Personal care and continuity of care are core values in Dutch general practice. GPs can work alone (solo practice), with two or more GPs (group practice), or be organized in larger health centres with several GPs and other healthcare provides. In their practice, GPs are supported by one or more medica9-10l receptionists and nurse practitioners. In general, medical receptionists are responsible for allocating patients to specific doctors; nurse practitioners are responsible for the care of patients with chronic diseases, elderly patients, or patients with psychosocial problems.

In the Netherlands, the three-year postgraduate GP training programme is offered by eight departments of general practice affiliated with academic medical centres. In their first and third years, trainees work in a general practice for four days a week. On the fifth day, trainees follow an educational programme at one of the departments. In the general practice, each GP trainee is supervised and coached by an experienced GP (supervisors). Most of the time,

For peer review only - http://bmjopen.bmj.com/site/about/guidelines.xhtml

trainees work independently but discuss their patients on a regular basis, or immediately if needed, with their designated supervisor. In their second year, trainees rotate through hospital, nursing home, and psychiatric care settings. GP supervisors, nurse practitioners, and medical receptionists affiliated with the GP Specialty Training Institute of the Amsterdam UMC, location AMC, are offered training days. Training days for GP supervisors are held multiple times a year and are compulsory. Training days for nurse practitioners and medical receptionists are held twice a year and are voluntary.

	**Design**	**Trainees (Y1/Y2/Y3)**	**Supervisors**	**Medical receptionist**	**Nurse practitioners**
**Recruitment**	Focus group	Multiple presentations during educational programme in 2016 & 2017	Email prior to training day June 2016 and presentation on training day April 2018	Presentation during training day March 2016	Email prior to training day April 2016
	Interview	By teachers of educational programme	NA	Presentation during training day March 2016	NA
**Location/Time**	Focus group	Regular education programme day at the department between May and June 2016	Training day for supervisors in June 2016 (2) and April 2018 (1)	After work hours at GP department	Training day April 2016
	Interview	Trainees' place of preference between January	NA	MRs' place of preference in December 2016*	NA

For peer review only - http://bmjopen.bmj.com/site/about/guidelines.xhtml

		and February 2017			
**Duration**	Focus group	40 minutes	60 minutes	60 minutes	60 minutes
	Interview	20 minutes	NA	30 minutes	NA

Table 1- Used recruitment methods and the number, place and duration of the focus groups or interview
* For logistic reasons, one medical receptionist was interviewed separately.

Participants

GP trainees, GP supervisors, medical receptionists, and nurse practitioners affiliated with the GP Specialty Training Institute of the Amsterdam UMC (location AMC) were invited to participate. All participants came from different general practices. See Table 1 for more detail on the number of participants, recruitment methods used, and the number, place, and duration of the focus groups or interviews. Prior to each focus group or interview, participants were given study information and informed consent was obtained. The study was approved by the ethics review board of the Dutch Association for Medical Education (file number 630). While all focus groups were homogenous with regard to profession, they were heterogeneous with regard to practice organization (single GP or group practice or health centre) and level of their trainee (first or third year) to stimulate discussion.

Data collection

Topic lists, covering the role of trust, supervision, patients' perspectives and preferences, continuity of care, and contextual factors (for example, practice size and number of employees), were used for the focus meetings and interviews and were continuously updated, based on findings. Focus groups were led by skilled moderators experienced in qualitative research and familiar with the scope of this study. The first author (SdB) observed most of the focus groups. If this was not possible for logistic or ethical reasons, another skilled observer

For peer review only - http://bmjopen.bmj.com/site/about/guidelines.xhtml

(SvR) familiar with the study protocol took over. All focus groups and interviews were audio-taped and transcribed verbatim. Transcripts were pseudonymized.

Data analysis

We used thematic analysis with a constant comparison methodology to guide our analysis(27). Initially two team members (SdB and SvR) independently coded two transcripts. After these first two transcripts, SdB and SvR discussed the initial coding scheme until they reached consensus. Next, five more transcripts were independently coded by SdB and SvR, using constant comparison with the earlier codes and adding new codes were needed. These codes were discussed in detail between SdB, SvR and MV until consensus was reached. Data from subsequent transcripts were further coded by SdB using the discussed codes scheme and, where needed, new codes were added and other code were merged or adopted. During this phase SdB, SvR and MR met on regular basis to discuss the code scheme and overarching themes. To fully explore the themes and their mutual relationship, multiple diagrams and figures were created. After proximally 13 transcripts the code scheme seemed sufficient to code the last transcripts, indicating data saturation. This final code scheme and the earlier figures were discussed within the whole research group. The research team included a GP trainee/PhD student (SdB) and four researchers, all experienced in medical education research and qualitative methodology: an educationalist and medical doctor (NvD), a cognitive psychologist (MV), a general practitioner (AK), and a research assistant/educationalist (SvR).

For peer review only - http://bmjopen.bmj.com/site/about/guidelines.xhtml

Results

There were 73 participants. Eleven focus groups were held, of which five with GP trainees (one with first-year trainees, two with second-year trainees, two with third-year trainees), three with supervisors, two with nurse practitioners, and two with medical receptionists. Additionally, to obtain a deeper understandings of our first findings, six interviews were held with third-year trainees. Demographics can be found in Table 2.

	**Trainees (n=37)**	**Supervisors (n=18)**	**Medical receptionists (n=6)**	**Nurse practitioners (n=12)**
**Age (median, range)**	30 (27-35)	56 (35-62)	53 (39-60)	53 (38-63)
**Gender (M/F)**	6 (16.2%) /31 (83.8%)	5 (27.8%) / 13 (72.2%)	0/6 (100%)	0/12 (100%)
**Practice organization**				
• **Single**	12 (32.4%)	5 (27.8%)	0	1 (8.3%)
• **Group**	16 (43.2%)	10 (55.6%)	5 (83.3%)	7 (58.3%)
• **Health care centre**	3 (21.6%)	3 (16.7%)	1 (16.6%)	4 (33.3%)
**Level of training (Y1/Y2/Y3)***	4 (10.8%) / 16 (43.2%) / 17 (45.9%)	NA	NA	NA
**Years as professional** (median, range)**	NA	23.5 (7–33)	15 (5–43)	14 (7–17)
**N of focus groups/interviews**	5/6	3/0	2/1	2/0
**Total participants in focus groups (range in each focus group)**	31 (5-8)	18 (5-7)	5(2-3)	12 (6)

Table 2 - Demographic data.
* only applicable for trainees. **only applicable for supervisors, MR and NP

In short

The allocation of patients to either a GP trainee or GP supervisor, and therefore trainees' patient mix, is influenced by several interacting factors. The relationship between doctor (supervisor or trainee) and patient in the context of the patient's health problem played an essential role. Trainees obviously did not have a trustful relationship with patients yet, but both they and team members could compensate for this in various ways. Whether this occurs in practice depended on three influencing factors: trainees' behaviour, attitudes of the team, and context (Fig 1). Below we will first present how the patient-doctor relationship affects

For peer review only - http://bmjopen.bmj.com/site/about/guidelines.xhtml

trainees' patient mix. Subsequently, we will explain how practices mayt overcome the lack in trusting relationship between trainee and patient, and how this process is influenced by trainees' behaviour, attitudes of the team and the context (influencing factors).

[**Figure 1** - Factors influencing the patient mix of general practitioner trainees]

Disease characteristics & doctor–patient relationship

According to our participants, a central factor affecting the patient mix of trainees was the doctor–patient relationship in relation to the patient's health problem. The interviewees noticed that patients generally preferred to see their 'own' doctor, because the doctor knew the patient and his/her (disease) history and social context and was trusted. However, the patient's preference could change depending on the perceived severity of their health problems. For minor ailments or urgent problems patients were prepared to see the trainee because quick access or timeliness became more important.

NP no. 415: If someone comes with, for instance, heart rhythm disturbances or chest pain, they are happy if they can be seen quickly and that can also be a trainee. But if they come with mental health problems, and their GP knows the family situation etc, then they prefer to see that doctor, because he/she knows about them and their situation

In general, our participants expressed it was felt that trainees were not in a practice long enough to really become the trusted doctor. This lack of a trainee–patient relationship seemed to affect the patient mix, especially when patients came with chronic and/or complex conditions.

For peer review only - http://bmjopen.bmj.com/site/about/guidelines.xhtml

Trainee no E1: Yes, I think that, this year, that I have had relatively little experience with chronic health problems and their treatment. If my supervisor is away a week, then it gets interesting. Otherwise they are mainly seen by my supervisor.

Overcoming the lack of doctor– patient relationship

Transference of trust

Findings showed that team members trusted by patients, such as the medical receptionist and the supervisor, could transfer this trust to the trainee, by emphasizing the trainee's benefits. For example trainees' previous work experience, pointing out that the trainee would have more time for them, and might have a new take on their problems. Medical receptionists in particular used these arguments if a patient was in doubt about who to see:

MR no 3: But sometimes if they've [the patient] had a problem for a long time, I'll say " Who knows, give it [red: seeing the trainee] a go, it may shed new light on the problem. And then they say "OK, I'll try it

This process could be strengthened by the presence of the supervisor or nurse practitioner during the first consultation of the trainee with the patient. If all went well, the patient could be transferred to the care of the trainee:

Trainee no 2: I had a patient who needed palliative care in the first 2 weeks of the year and then my supervisor said to the patient], when we were at his bedside "Look, I trust [name of trainee], it's OK. I have to go to a conference; he'll take over". I hardly knew the patient but he said " OK, we'll do that" And everything went OK. But because he [red: the supervisor] made it explicit at that moment, it took away a lot of worry and stress.

For peer review only - http://bmjopen.bmj.com/site/about/guidelines.xhtml

Acquisition of trust

Although trainees obviously did not immediately have a trustful doctor–patient relationship, this could develop rapidly, often after a successful first encounter.

MR no 1: But if they [patients] have seen [a trainee] once, perhaps because their 'own' doctor couldn't see them, and that goes well, then there is no problem with subsequent appointments.

Whether the trainee gained the patient's trust and developed a good patient–doctor relationship depended to a large extent on the trainee's behaviour (see below).

Influencing factors

Trainees' behaviour

Trainees could affect their patient mix directly or indirectly. Directly, for example, if they invested time and energy in building a relationship with their patients, trainees could become the preferred doctor.

Trainee nr E2: I had a female patient with a BIRADS-4 and I had to tell her. And that was population screening. She started treatment. I phoned her every 2 weeks. She said "I appreciate you phoning to ask how things are going". I try to do that sort of thing. Yes, I think you can gain people's trust in that way.

Trainees could influence their patient mix more indirectly by creating a supportive work environment. As stated earlier, trainees are dependent on other team members when it comes

For peer review only - http://bmjopen.bmj.com/site/about/guidelines.xhtml

to patient allocation or transfer. However, these team members first need to feel that they can trust the trainee before they will allocate patients to the trainee. Trainees could influence this in several ways, one of which was being keen, as explained by the following medical receptionist who was asked what trainees can do to speed up trust:

MR3: Well, by mentioning it – if you have patients with this or that problem, let me see them. Some trainees do this. I can't think of anything specific at the moment, but if they say "I find that interesting, you can send them to me"

Trainees should also try to establish a good relationship with other staff, as explained below:

Supervisor no 17: What also may help, especially in the beginning of their training year, is that, if a trainee does not have a full surgery, that they do not stay in their room, but go to the medical receptionist. Personal contact between receptionists and trainees. The receptionist will work harder for the trainee, if they have a good relationship..

Trainees should also try to involve other staff in their training and learning, because this made staff more willing to allocate patients with a specific problem to them, as the following examples show:

MR no 2: Yes, If I know from this or that, hat she needs to discuss it at the educational day at the institute this week. I'll indeed try to plan it.

Attitude of team members

First encounters between patient and trainee are mainly arranged by supervisors, medical receptionists, and nurse practitioners. However, team members sometimes struggled with achieving a balance between teaching the trainee and providing good patient care, which could

For peer review only - http://bmjopen.bmj.com/site/about/guidelines.xhtml

affect the trainee patient mix. This is illustrated by the following conversation between two supervisors:

Supervisor no 2: I sometimes find it difficult. Recently, a woman phoned for me when I wasn't at the practice. She has recurrent lung cancer. The trainee took the call. Should I take over? should I contact the patient? Who should be in charge. I find it difficult.

Supervisor no 5:What do you find difficult?

Supervisor no 2:You don't want to let the patient go, you want the patient to know that you're there for her. On the other hand, she [the trainee] should also get the chance to do things.

The feeling of being involved in the trainee's education was important. As shown above, the nurse practitioner and medical receptionist were more willing to take the trainee's learning objectives into consideration when allocating a patient if they felt involved in that trainee's education. However, not every team member was equally involved or wanted to be involved.

Context

Our participants noticed that the context has also an important influence. For example, every practice has their own contextual factors such as the availability of a nurse practitioner, the organization and location of a practice, the size of a team, the number of previous trainees, and so on. An example of a contextual factor (having a walk-in surgery) that influenced trainee patient mix is explained by a supervisor:

Supervisor no 10: The walk-in surgery is always run by trainees in this practice. They can learn about minor acute problems but also make contact with patients, make an follow-up appointment with them. In this way you can build up your own patient list in no time. It's very satisfactory and works well.

For peer review only - http://bmjopen.bmj.com/site/about/guidelines.xhtml

Context also seemed to affected strength of the doctor–patient relationship. If a general practice has only one doctor, then the doctor–patient relationship is probably strong and patients will be less willing to consult a trainee. This in contrast with larger practices where patients rotate between doctors, whereby patients have less strong ties with a single doctor.

Trainee no 15: I worked in a solo practice with a 64-year-old GP who had worked there for 34 years. He was THE doctor. So yes, it's probably good to gain experience in a health centre with eight GPs and three trainees, but whether it makes a difference to the patients? I don't know. In a solo practice the trusting relationship is very sure and resistant, certainly if the doctor has been there for long ... Maybe somebody here has experience with this? To be in a health center with multiple doctors?

Trainee no 16: Yes, I think that you [the doctor] will be more replaceable. When a patient always sees the same GP, than he always wants to go there. If there are at least 6 doctors it [the patient] would be more flexible, so I think it matters.

For peer review only - http://bmjopen.bmj.com/site/about/guidelines.xhtml

Discussion

Summary of main findings

The mix of patients GP trainees see is shaped by a several factors. The characteristics of the health problem and the bond between trainee and patient are major determinants of this mix. Patients with complex problems tend to prefer their 'own doctor', because they trust him/her. It is generally recognized that a strong, trustful doctor–patient relationship is essential for the provision of high-quality care. Trainees cannot have such a relationship, especially not at the start of their training year in a new general practice. Our findings suggest that trainees and team members can take steps to surmount this lack of a trustful relationship. Team members can provide trainees with opportunities to see specific patients, and trainees can take specific steps to develop trustful doctor–patient relationships. Whether this happens depends on trainee behaviour, the attitudes of team members, and the context of the general practice. Team members have to trust the trainee before they are willing to allocate or transfer patients with complex medical conditions to the trainee. This is influenced by both practice culture and the trainee's behaviour. Trainees should invest in a good relationship with team members involve them in their development and needs, and show a keenness to learn. Trainees should also invest time and energy in building a relationship with patients, by being pro-active. This will help patients to trust them. In this way, trainees will generate their own list of patients with diverse medical problems of varying complexity. By doing so, they deliberately influence their own learning process in a positive way.

Strengths & Limitations

In this study we took a broad point of view and incorporate the perspectives from multiple participants with different roles within the GP practice. Although we did not incorporate

For peer review only - http://bmjopen.bmj.com/site/about/guidelines.xhtml

patients' perspectives, findings from previous studies on patients' perspectives indicate that their views overlap with our(8, 28, 29). The use of qualitative methods enabled us to look in depth at factors affecting the patient mix of GP trainees from different perspectives. The fact that our findings are based on the patient mix of a Dutch GP trainee could be regarded as a limitation for translating our findings to other settings. However, the importance of continuity of care, especially for patients with chronic conditions, is acknowledged overall (28, 30-32). So this factor will probably influence the patients seen by GP trainees everywhere. However, it is less likely that our findings can be translated to a hospital setting, where the strong doctor–patient relationship seen in primary care is less likely to exist, because doctor–patient contact there is more fleeting.

Comparison with previous research and theory

A body of knowledge exists about the continuity of care, its relationship with the complexity of disease, and the high value patients and doctors place on it(29, 30, 33). However, only a few studies have investigated this in relation to the mix of patients seen by GP trainees, and these studies have tended to take the patients' point of view as outcome(8, 15, 29, 34). Although we had a different perspective, our findings are in line with this research. Bonney et al. found that older patients have a strong preference for their 'own' GP, and that their attitude towards the GP trainee has to be viewed within this perspective(34). This finding is consistent with multiple cross-sectional studies from different countries(10, 28, 29, 35).

However, not only patients' preferences influenced the trainees' patient mix. We found that supervisors, nurse practitioners, and medical receptionists also have their influence. This finding mirrors those of Bannister et al., who found that nurses and other team members in a hospital setting can hinder paediatric trainees in their learning opportunities(6). Our finding of trainees as important influencers is consistent with the findings of multiple studies on how

For peer review only - http://bmjopen.bmj.com/site/about/guidelines.xhtml

trainees learn. Among others, previous research has found that learner engagement is important for trainees to practise skills(6), to create learning opportunities(36), and to create a more learning-oriented environment (37, 38).

Our findings are consistent with socio-cultural theories about learning(39). The situated learning theory of Lave and Wenger emphasizes the importance of participation in daily work to learning(40). Learners are active participants, and through participation they become full members of a 'community of practice'. Newcomers evolve through engagement with their workplace and gain more responsibilities. We found that GP trainees need to engage in their communities: by actively participating and building work relationships, trainees will gain trust and gradually be given more responsibility. Trainees evolve then from a 'legitimate peripheral participator' into a legitimate member of the community. We found that trainees who made an effort and invested time in their work environment were important influencers of their learning and patient mix. This finding is consistent with earlier work by Billet and Bandura, who stated that learners have to engage to reach the full educational potential a workplace has to offer(17, 41).

Implications for research and practice

The mix of patients seen by GP trainees should offer trainees the opportunities they need to develop competencies and to acquire skills. Nonetheless, this study showed that the patient mix is affected by many, entangled factors. We found that greater trainee engagement with their workplace positively affected their patient mix. In a future study, we will investigate patients' perspectives on trainee involvement. Taken together, findings will provide information about how to optimize workplace learning in general practice. The findings will be used to create an educational intervention to enhance that GP trainees see a more learning-oriented mix of patients.

For peer review only - http://bmjopen.bmj.com/site/about/guidelines.xhtml

For peer review only

References

1. Teunissen PW, Scheele F, Scherpbier AJ, et al. How residents learn: qualitative evidence for the pivotal role of clinical activities. Med Educ. 2007;41(8):763-70.

2. Sagasser MH, Kramer AWM, Fluit C, et al. Self-entrustment: how trainees' self-regulated learning supports participation in the workplace. Adv Health Sci Educ Theory Pract. 2017;22(4):931-49.

3. Ericsson KA. Deliberate practice and the acquisition and maintenance of expert performance in medicine and related domains. Acad Med. 2004;79(10 Suppl):S70-81.

4. Ericsson KA. Acquisition and Maintenance of Medical Expertise: A Perspective From the Expert-Performance Approach With Deliberate Practice. Acad Med. 2015;90(11):1471-86.

5. Ten Cate O, Gruppen LD, Kogan JR, et al. Time-Variable Training in Medicine: Theoretical Considerations. Acad Med. 2018;93(3S Competency-Based, Time-Variable Education in the Health Professions):S6-S11.

6. Bannister SL, Dolson MS, Lingard L, et al. Not just trust: factors influencing learners' attempts to perform technical skills on real patients. Med Educ. 2018.

7. de Jong J, Visser MR, Wieringa-de Waard M. Steering the patient mix of GP trainees: results of a randomized controlled intervention. Med Teach. 2013;35(2):101-8.

8. Bonney A, Phillipson L, Reis S, et al. Patients' attitudes to general practice registrars: a review of the literature. Educ Prim Care. 2009;20(5):371-8.

9. Bonney A, Jones SC, Iverson D, et al. Trust, continuity and agency: keys to understanding older patients' attitudes to general practice trainees. Educ Health (Abingdon). 2014;27(1):39-46.

For peer review only

10. Nakar S, Levi D, Rosenberg R, et al. Patient attitudes to being treated by junior residents in the community. Patient Educ Couns. 2010;78(1):111-6.

11. Mol SS, Peelen JH, Kuyvenhoven MM. Patients' views on student participation in general practice consultations: a comprehensive review. Med Teach. 2011;33(7):e397-400.

12. Sagasser MH, Kramer AW, van der Vleuten CP. How do postgraduate GP trainees regulate their learning and what helps and hinders them? A qualitative study. BMC Med Educ. 2012;12:67.

13. van der Zwet J, Zwietering PJ, Teunissen PW, et al. Workplace learning from a socio-cultural perspective: creating developmental space during the general practice clerkship. Adv Health Sci Educ Theory Pract. 2011;16(3):359-73.

14. Sagasser MH, Kramer AW, van Weel C, et al. GP supervisors' experience in supporting self-regulated learning: a balancing act. Adv Health Sci Educ Theory Pract. 2015;20(3):727-44.

15. Hauer KE, Ten Cate O, Boscardin C, et al. Understanding trust as an essential element of trainee supervision and learning in the workplace. Adv Health Sci Educ Theory Pract. 2014;19(3):435-56.

16. Wearne S, Dornan T, Teunissen PW, et al. General practitioners as supervisors in postgraduate clinical education: an integrative review. Med Educ. 2012;46(12):1161-73.

17. Billett S. Learning through health care work: premises, contributions and practices. Med Educ. 2016;50(1):124-31.

18. Chen HC, Teherani A. Workplace affordances to increase learner engagement in the clinical workplace. Med Educ. 2015;49(12):1184-6.

19. Berkhout JJ, Helmich E, Teunissen PW, et al. Context matters when striving to promote active and lifelong learning in medical education. Med Educ. 2018;52(1):34-44.

For peer review only

For peer review only - http://bmjopen.bmj.com/site/about/guidelines.xhtml

20. Deketelaere A, Kelchtermans G, Struyf E, et al. Disentangling clinical learning experiences: an exploratory study on the dynamic tensions in internship. Med Educ. 2006;40(9):908-15.

21. Stok-Koch L, Bolhuis S, Koopmans R. Identifying factors that influence workplace learning in postgraduate medical education. Educ Health (Abingdon). 2007;20(1):8.

22. van de Wiel MW, Van den Bossche P, Janssen S, et al. Exploring deliberate practice in medicine: how do physicians learn in the workplace? Adv Health Sci Educ Theory Pract. 2011;16(1):81-95.

23. Hauer KE, Oza SK, Kogan JR, et al. How clinical supervisors develop trust in their trainees: a qualitative study. Med Educ. 2015;49(8):783-95.

24. Sterkenburg A, Barach P, Kalkman C, et al. When do supervising physicians decide to entrust residents with unsupervised tasks? Acad Med. 2010;85(9):1408-17.

25. Stalmeijer RE, McNaughton N, Van Mook WN. Using focus groups in medical education research: AMEE Guide No. 91. Med Teach. 2014;36(11):923-39.

26. Faber MJ, Burgers JS, Westert GP. A sustainable primary care system: lessons from the Netherlands. J Ambul Care Manage. 2012;35(3):174-81.

27. Braun V, Clarke V. Using thematic analysis in psychology. Qualitative Research in Psychology. 2006;3:77-101.

28. Malcolm CE, Wong KK, Elwood-Martin R. Patients' perceptions and experiences of family medicine residents in the office. Can Fam Physician. 2008;54(4):570-1, 1 e1-6.

29. Pearlman J, Moran SL, Van Driel M, et al. Continuity of care in general practice vocational training: prevalence, associations and implications for training. Education for Primay Care. 2016;27(2):27-36.

For peer review only

30. Haggerty JL, Roberge D, Freeman GK, et al. Experienced continuity of care when patients see multiple clinicians: a qualitative metasummary. Ann Fam Med. 2013;11(3):262-71.

31. Mainous AG, 3rd, Baker R, Love MM, et al. Continuity of care and trust in one's physician: evidence from primary care in the United States and the United Kingdom. Fam Med. 2001;33(1):22-7.

32. Merriel SW, Salisbury C, Metcalfe C, et al. Depth of the patient-doctor relationship and content of general practice consultations: cross-sectional study. Br J Gen Pract. 2015;65(637):e545-51.

33. Ridd MJ, Lewis G, Peters TJ, et al. Patient-doctor depth-of-relationship scale: development and validation. Ann Fam Med. 2011;9(6):538-45.

34. Bonney A, Phillipson L, Jones SC, et al. Older patients' attitudes to general practice registrars - A qualitative study. Aust Fam Physician. 2009;38(11):927-31.

35. Crawford GH, Gutman A, Kantor J, et al. Patients' attitudes toward resident participation in dermatology outpatient clinics. J Am Acad Dermatol. 2005;53(4):710-2.

36. Berkhout JJ, Helmich E, Teunissen PW, et al. Exploring the factors influencing clinical students' self-regulated learning. Med Educ. 2015;49(6):589-600.

37. Nothnagle M, Anandarajah G, Goldman RE, et al. Struggling to be self-directed: residents' paradoxical beliefs about learning. Acad Med. 2011;86(12):1539-44.

38. Goldman E, Plack M, Smith J, et al. Learning in a chaotic enviroment. Journal of Workplace Learning. 2009;21(7):555-74.

39. Mann K, Dornan T, Teunissen PW. Perspectives on learning. In: Dornan T, Mann KV, Scherpbier A, et al., editors. Medical Education: Theory and Practice: Elsevier; 2011. p. 17-38.

For peer review only - http://bmjopen.bmj.com/site/about/guidelines.xhtml

40. Lave J, Wenger E. Situated Learning: legitimate pheripheral participation. Cambridge UK: Canbridge University Press; 1991 1991.

41. Bandura A. Social cognitive theory: an agentic perspective. Annu Rev Psychol. 2001;52:1-26.

For peer review only

For peer review only - http://bmjopen.bmj.com/site/about/guidelines.xhtml

Acknowledgements

The authors wish to thank all participants who were willing to share their thoughts about trainees' patient mix, as well as the staff of the GP specialty training of the Amsterdam UMC (location AMC) who helped us with the organisation of the focus groups and interviews.

Funding statement

This work is funded by a grant from ZonMW (grant number: 839130001)

Conflict of interest

We declare no competing interest

Authors Statement

SdB, MV and NvD designed the study. SdB, SvR and MV performed the interviews and analysis with support from NvD and AK. All authors discussed the results. SdB wrote the manuscript with input from all authors

.

Data sharing

The data may be obtained from the authors upon request

Patient and Public Involvement

We did not involve patient or the public. The research question was based on previous studies and part of a larger, peer-reviewed study protocol. The results will be disseminated through training programs for GP trainers and trainees. We developed workshops in which trainers and trainees are informed about the main result of our study, and are taught skills to influence

For peer review only

their patient mix. These workshops are part of further investigation and on trainees' patient mix and are implemented within the GP specialty training program of the AMC.

For peer review only

For peer review only - http://bmjopen.bmj.com/site/about/guidelines.xhtml

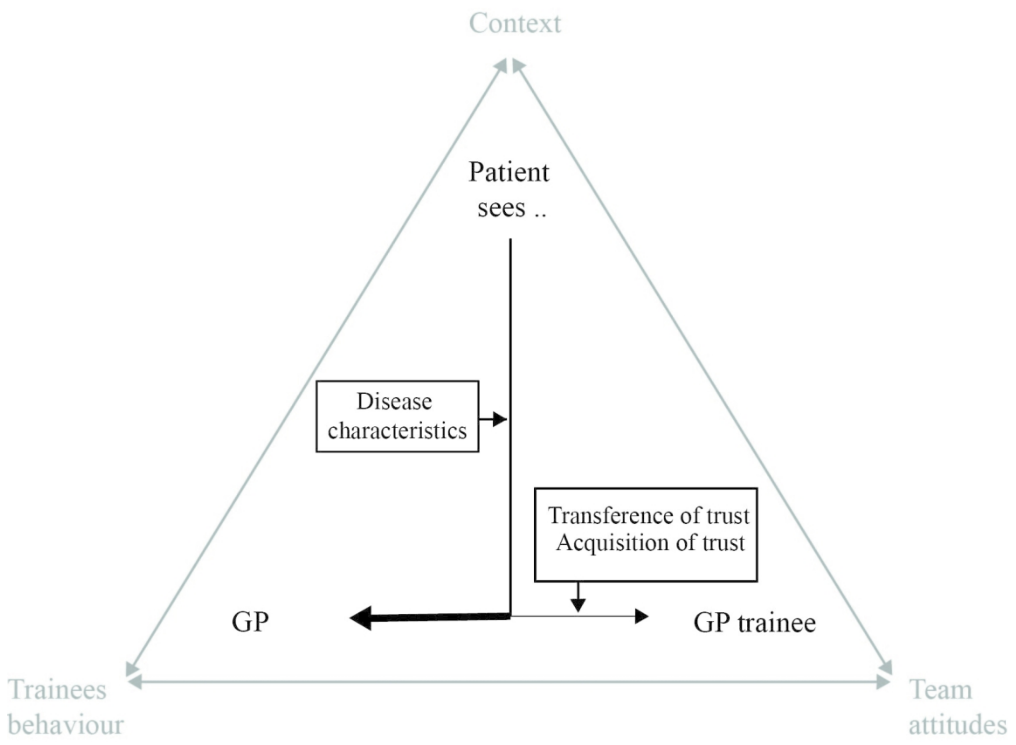

Figure 1 - Factors influencing the patient mix of general practitioner trainees

144x106mm (300 x 300 DPI)

Reporting checklist for qualitative study.

Based on the SRQR guidelines.

Instructions to authors

Complete this checklist by entering the page numbers from your manuscript where readers will find each of the items listed below.

Your article may not currently address all the items on the checklist. Please modify your text to include the missing information. If you are certain that an item does not apply, please write "n/a" and provide a short explanation.

Upload your completed checklist as an extra file when you submit to a journal.

In your methods section, say that you used the SRQRreporting guidelines, and cite them as:

O'Brien BC, Harris IB, Beckman TJ, Reed DA, Cook DA. Standards for reporting qualitative research: a synthesis of recommendations. Acad Med. 2014;89(9):1245-1251.

		Reporting Item	Page Number
**Title**			
	#1	Concise description of the nature and topic of the study identifying the study as qualitative or indicating the approach (e.g. ethnography, grounded theory) or data collection methods (e.g. interview, focus group) is recommended	1

For peer review only

Abstract

	#2	Summary of the key elements of the study using the abstract format of the intended publication; typically includes background, purpose, methods, results and conclusions	2

Introduction

Problem formulation	#3	Description and signifcance of the problem / phenomenon studied: review of relevant theory and empirical work; problem statement	5
Purpose or research question	#4	Purpose of the study and specific objectives or questions	6

Methods

Qualitative approach and research paradigm	#5	Qualitative approach (e.g. ethnography, grounded theory, case study, phenomenolgy, narrative research) and guiding theory if appropriate; identifying the research paradigm (e.g. postpositivist, constructivist / interpretivist) is also recommended; rationale. The rationale should briefly discuss the justification for choosing that theory, approach, method or technique rather than other options available; the assumptions and limitations implicit in those choices and how those choices influence study conclusions and transferability.	10

For peer review only

		As appropriate the rationale for several items might be discussed together.	
Researcher characteristics and reflexivity	#6	Researchers' characteristics that may influence the research, including personal attributes, qualifications / experience, relationship with participants, assumptions and / or presuppositions; potential or actual interaction between researchers' characteristics and the research questions, approach, methods, results and / or transferability	10
Context	#7	Setting / site and salient contextual factors; rationale	7
Sampling strategy	#8	How and why research participants, documents, or events were selected; criteria for deciding when no further sampling was necessary (e.g. sampling saturation); rationale	7-9
Ethical issues pertaining to human subjects	#9	Documentation of approval by an appropriate ethics review board and participant consent, or explanation for lack thereof; other confidentiality and data security issues	9
Data collection methods	#10	Types of data collected; details of data collection procedures including (as appropriate) start and stop dates of data collection and analysis, iterative process, triangulation of sources / methods, and modification of procedures in response to evolving study findings; rationale	8-10

For peer review only

Data collection instruments and technologies	#11	Description of instruments (e.g. interview guides, questionnaires) and devices (e.g. audio recorders) used for data collection; if / how the instruments(s) changed over the course of the study	9-10
Units of study	#12	Number and relevant characteristics of participants, documents, or events included in the study; level of participation (could be reported in results)	11
Data processing	#13	Methods for processing data prior to and during analysis, including transcription, data entry, data management and security, verification of data integrity, data coding, and anonymisation / deidentification of excerpts	10
Data analysis	#14	Process by which inferences, themes, etc. were identified and developed, including the researchers involved in data analysis; usually references a specific paradigm or approach; rationale	10
Techniques to enhance trustworthiness	#15	Techniques to enhance trustworthiness and credibility of data analysis (e.g. member checking, audit trail, triangulation); rationale	10
**Results/findings**			
Syntheses and interpretation	#16	Main findings (e.g. interpretations, inferences, and themes); might include development of a theory or model, or integration with prior research or theory	11-12

For peer review only

Links to empirical data	#17	Evidence (e.g. quotes, field notes, text excerpts, photographs) to substantiate analytic findings	11-17
**Discussion**			
Intergration with prior work, implications, transferability and contribution(s) to the field	#18	Short summary of main findings; explanation of how findings and conclusions connect to, support, elaborate on, or challenge conclusions of earlier scholarship; discussion of scope of application / generalizability; identification of unique contributions(s) to scholarship in a discipline or field	18-20
Limitations	#19	Trustworthiness and limitations of findings	18-19
**Other**			
Conflicts of interest	#20	Potential sources of influence of perceived influence on study conduct and conclusions; how these were managed	26
Funding	#21	Sources of funding and other support; role of funders in data collection, interpretation and reporting	26

The SRQR checklist is distributed with permission of Wolters Kluwer © 2014 by the Association of American Medical Colleges. This checklist was completed on 06. June 2019 using https://www.goodreports.org/, a tool made by the EQUATOR Network in collaboration with Penelope.ai

For peer review only

BMJ Open

Professionals' perspectives on factors affecting GP trainees' patient mix: Results from an interview and focus group study among professionals working in Dutch general practice.

Journal:	*BMJ Open*
Manuscript ID	bmjopen-2019-032182.R1
Article Type:	Original research
Date Submitted by the Author:	19-Sep-2019

Complete List of Authors:	de Bever, Sarah; Amsterdam UMC - Locatie AMC, Department of General Practice/ GP Specialty Training Program<br>van Rhijn, Suzanne; Amsterdam UMC - Locatie AMC, Department of General Practice/ GP Specialty Training Program<br>van Dijk, Nynke; Amsterdam UMC - Locatie AMC, Department of General Practice/GP Specialty Training<br>Kramer, Anneke ; Leiden University Medical Center, Department of Public Health and Primary Care<br>Visser, Mechteld; Amsterdam UMC - Locatie AMC, Department of General Practice/ GP Specialty Training

**Primary Subject Heading**:	Medical education and training
Secondary Subject Heading:	General practice / Family practice
Keywords:	MEDICAL EDUCATION & TRAINING, PRIMARY CARE, QUALITATIVE RESEARCH

SCHOLARONE™ Manuscripts

Professionals' perspectives on factors affecting GP trainees' patient mix: Results from an interview and focus group study among professionals working in Dutch general practice.

Authors: Sarah de Bever (MD)[a] , Suzanne C. van Rhijn (BSc)[a], Nynke van Dijk (MD, PhD)[a], Anneke W.M. Kramer (MD, PhD)[b], Mechteld R.M. Visser (PhD)[a]

Affliations:
a : Amsterdam UMC, University of Amsterdam, Department of General Practice/ GP Specialty Training Program, Amsterdam Public Health Research Institute, Amsterdam, Netherlands
b: Department of Public Health and Primary Care, Leiden University Medical Centre, Leiden, the Netherlands

Corresponding author: s.debever@amsterdamumc.nl. Adress: Meibergdreef 15, room J2-113, 1105 AZ Amsterdam, the Netherlands

Contact information:
Suzanne C. van Rhijn: suzannevanrhijn@hotmail.com
Nynke van Dijk: n.vandijk@amc.uva.nl
Anneke W.M. Kramer: a.w.m.kramer@lumc.nl
Mechteld. R.M. Visser: m.r.visser@amc.uva.nl

Word count: 3813

For peer review only

For peer review only - http://bmjopen.bmj.com/site/about/guidelines.xhtml

Abstract

Introduction: Seeing and treating patients in daily practice forms the basis of general practitioner (GP) training. However, the types of patients seen by GP trainees do not always match trainees' educational needs. Knowledge about factors that shape the mix of patient types is limited, especially with regard to the role of the professionals who work in the GP practice.

Aim: We investigated factors affecting the mix of patients seen by GP trainees from the perspective of professionals.

Design & Setting: This qualitative study involved GP trainees, GP supervisors, medical receptionists, and nurse practitioners affiliated with a GP Specialty Training Institute in the Netherlands.

Methods: Twelve focus groups and seven interviews with 73 participants were held. Data collection and analysis were iterative, using thematic analysis with a constant comparison methodology.

Results: The characteristics of patients' health problems and the bond between the doctor and patient are important determinants of GP trainees' patient mix. Because trainees have not yet developed bonds with patients, they are less likely to see patients with complex health problems. However, trainees can deliberately influence their patient mix by paying purposeful attention to bonding with patients and by gaining professional trust through focused engagement with their colleagues.

Conclusion: Trainees' patient mix is affected by various factors. Trainees and team members can take steps to ensure that this mix matches trainees' educational needs, but their success depends on the interaction between trainees' behavior, the attitudes of team members, and the

For peer review only - http://bmjopen.bmj.com/site/about/guidelines.xhtml

context. The findings show how the mix of patients seen by trainees can be influenced to become more trainee-centered and learning-oriented.

Keywords*:* *Postgraduate Medical Education, General Practice, Doctor-Patient Relationship. Workplace-Based Learning, Trust, Patient mix*

For peer review only

Article summary – strengths & limitations of the study

- We conducted focus groups and interviews with 73 participants who together represented all professional roles in general practice (GP, medical receptionist, nurse practitioner, GP trainee).
- Qualitative analysis allowed for an in-depth exploration of factors affecting trainees' patient mix.
- Combining all these perspectives, this study provides a rich insight into factors that affect trainees' patient mix.
- While patients' perspectives are missing from this study, literature on their perspectives suggest similar findings.
- The results are most applicable to settings in which continuity of care is important.

For peer review only

Introduction

Seeing and treating patients forms the basis of training for medical graduates specializing to become general practitioners (GPs)(1, 2). Thus their development of competence and skills depends on the mix of patients seen. According to Ericsson's theory of deliberate practice, mastery of competencies is achieved through repetitive practice, monitoring, and timely feedback from a trusted coach followed by further practice(3, 4). In the clinical workplace setting, this means repetitive cycles of the same patient symptoms or situations. Yet in everyday practice the mix of patients seen by GP trainees cannot always be organized in this way(5).The mix seems to be affected by a number of factors other than the educational needs of the trainee(6, 7). To make GP training more effective, a thorough understanding of all factors affecting the patient mix of GP trainees is needed.

Previous studies of trainees' patient mix mostly focused on patients' willingness to consult the trainee(8-11). However, trainees, their supervisors, and the practice environment probably also affect the patient mix. It has been found, for example, that GP trainees influence their patient mix in order to fill their knowledge gaps(12), but this requires developmental space(2, 13). Supervisors can provide this space, however they need to trust the trainee first(14-16). The relationship between GP trainee and supervisor affects the trainee's ability to steer his/her learning, and therefore probably also their patient mix(2, 12). Contextual factors, such as the work climate, may also influence which patients are seen by the trainee. While trainee workplaces should 'invite and support' learning(17-19), some are more work-oriented than supportive for learners(6, 20). For example, medical receptionists are responsible for allocating patients and they may not necessarily prioritize a trainee's educational needs when allocating patients. Many studies have investigated how trainees learn(1, 12, 21, 22), how supervisors develop their trust(23, 24), the importance of work relationships, and the

For peer review only - http://bmjopen.bmj.com/site/about/guidelines.xhtml

influence of context on learning(2, 12, 19), but none have looked at the impact of the trainee's patient mix. Yet this information is needed in order to support training in the general practice. In this study, we investigated factors influencing the patient mix of GP trainees. To this end, we carried out a qualitative study involving different health professionals (GP trainees, their supervisors, medical receptionists, and nurse practitioners) in a general practice setting. Findings can be used to ensure that trainees see a mix of patients appropriate to their needs, thereby optimizing workplace-based learning.

For peer review only

For peer review only - http://bmjopen.bmj.com/site/about/guidelines.xhtml

Methods

Design

Focus group meetings and additional semi-structured interviews were used in this qualitative study. Focus groups were chosen because they provide the opportunity for in-depth investigation of a topic through group discussion(25).

Participants

GP trainees, GP supervisors, medical receptionists, and nurse practitioners affiliated with the GP Specialty Training Institute of the Amsterdam UMC (location AMC) were invited to participate. All participants came from different general practices. See Table 1 for more details regarding the number of participants; recruitment methods used; and the number, place, and duration of the focus groups or interviews. After the initial analysis, we held six interviews with trainees to deepen our first impressions. We conducted purposive sampling on final year trainees, because they appeared to be the most informative participants.. While all focus groups were homogenous with regard to profession we ensured that they were heterogeneous with regard to practice organization (single practice, group practice, or health center) and level of the trainee to stimulate discussion.

	**Design**	**Trainees (Y1/Y2/Y3)**	**Supervisors**	**Medical receptionist**	**Nurse practitioners**
**Recruitment**	Focus group	Multiple presentations during educational programme in 2016 & 2017	Email prior to training day June 2016 and presentation on training day April 2018	Presentation during training day March 2016	Email prior to training day April 2016

For peer review only - http://bmjopen.bmj.com/site/about/guidelines.xhtml

	Interview	By teachers of educational programme	NA	Presentation during training day March 2016	NA
**Location/Time**	Focus group	Regular education programme day at the department between May and June 2016	Training day for supervisors in June 2016 (2) and April 2018 (1)	After work hours at GP department	Training day April 2016
	Interview	Trainees' place of preference between January and February 2017	NA	MRs' place of preference in December 2016*	NA
**Duration**	Focus group	40 minutes	60 minutes	60 minutes	60 minutes
	Interview	20 minutes	NA	30 minutes	NA

Table 1- Recruitment methods and number, place and duration of the focus groups or interviews
* For logistic reasons, one medical receptionist was interviewed separately.

Setting

In the Netherlands, all residents are registered with a general practice. GPs are generalists, gatekeepers for specialist and hospital care, and patients' first contact with the healthcare system (26). Most general practices are in the community, so there is often a strong, long-standing relationship between GPs and patients (26). Personal care and continuity of care are core values in Dutch general practice. GPs can work alone (solo practice), with two or more GPs (group practice), or be organized in larger health centers with several GPs and other healthcare provides. In their practice, GPs are supported by one or more medical receptionists

For peer review only - http://bmjopen.bmj.com/site/about/guidelines.xhtml

and nurse practitioners. In general, medical receptionists are responsible for allocating patients to specific doctors, while nurse practitioners are responsible for the care of patients with chronic diseases, elderly patients, or patients with psychosocial problems.

In the Netherlands, the three-year postgraduate GP training program is offered by eight departments of general practice affiliated with academic medical centers. In their first and third years, trainees work in a general practice four days each week. On the fifth day, trainees follow an educational program at one of the departments. In the general practice, each GP trainee is supervised and coached by an experienced GP (supervisor). Most of the time, trainees work independently but discuss their patients on a regular basis, or immediately if needed, with their designated supervisor. In their second year, trainees rotate through hospital, nursing home, and psychiatric care settings. GP supervisors, nurse practitioners, and medical receptionists affiliated with the GP Specialty Training Institute of the Amsterdam UMC, location AMC, are offered training days. Training days for GP supervisors are held multiple times a year and are compulsory. Training days for nurse practitioners and medical receptionists are held twice a year and are voluntary.

Patient and Public Involvement

We did not involve patients nor the public in this study. The research question was based on previous studies and was part of a larger, peer-reviewed study protocol. The results will be disseminated through training programs for GP supervisors and trainees. We developed workshops in which supervisors and trainees are informed about the main results of our study and are taught skills to influence their patient mix. These workshops are part of further investigations on trainees' patient mix and are implemented within the GP specialty training program of the AMC.

For peer review only - http://bmjopen.bmj.com/site/about/guidelines.xhtml

Data collection

Topic lists, covering the role of trust, supervision, patients' perspectives and preferences, continuity of care, and contextual factors (for example, practice size and number of employees), were used for the focus meetings and interviews and were continuously updated based on findings. Focus groups were led by skilled moderators experienced in qualitative research and familiar with the scope of this study. The first author (SdB) observed most of the focus groups. If this was not possible for logistic or ethical reasons, another skilled observer (SvR) familiar with the study protocol took over. All focus groups and interviews were audio-taped and transcribed verbatim. Transcripts were pseudonymized.

Data analysis

We used thematic analysis with a constant comparison methodology to guide our analysis(27). Initially two team members (SdB and SvR) independently coded two transcripts. After these first two transcripts, SdB and SvR discussed the initial coding scheme until they reached consensus. Next, five more transcripts were independently coded by SdB and SvR, using constant comparison with the earlier codes and adding new codes were needed. These codes were discussed in detail between SdB, SvR and MV until consensus was reached. Data from subsequent transcripts were further coded by SdB using the discussed codes scheme and, where needed, new codes were added and other code were merged or adopted. During this phase SdB, SvR and MR met on regular basis to discuss the code scheme and overarching themes. To fully explore the themes and their mutual relationship, multiple diagrams and figures were created. After proximally 13 transcripts the code scheme seemed sufficient to code the last transcripts, indicating data saturation. This final code scheme and the earlier figures were discussed within the whole research group. The research team included a GP trainee/PhD student (SdB) and four researchers, all experienced in medical education research

For peer review only - http://bmjopen.bmj.com/site/about/guidelines.xhtml

and qualitative methodology: an educationalist and medical doctor (NvD), a cognitive psychologist (MV), a general practitioner (AK), and a research assistant/educationalist (SvR).

Ethics approval

Prior to each focus group or interview, participants were given study information and informed consent was obtained. The study was approved by the ethics review board of the Dutch Association for Medical Education (file number 630).

For peer review only

Results

In total, eleven focus groups were held, of which five with GP trainees (one with first-year trainees, two with second-year trainees, two with third-year trainees), three with supervisors, two with nurse practitioners, and two with medical receptionists. Additionally, six interviews were held with third-year trainees before data saturation was reached. In total, we included 73 participants. The participants' demographics can be found in Table 2.

	**Trainees (n=37)**	**Supervisors (n=18)**	**Medical receptionists (n=6)**	**Nurse practitioners (n=12)**
**Age (median, range)**	30 (27-35)	56 (35-62)	53 (39-60)	53 (38-63)
**Gender (M/F)**	6 (16.2%) /31 (83.8%)	5 (27.8%) / 13 (72.2%)	0/6 (100%)	0/12 (100%)
**Practice organization**<br>• **Single**<br>• **Group**<br>• **Health care centre**	 12 (32.4%)<br>16 (43.2%)<br>3 (21.6%)	 5 (27.8%)<br>10 (55.6%)<br>3 (16.7%)	 0<br>5 (83.3%)<br>1 (16.6%)	 1 (8.3%)<br>7 (58.3%)<br>4 (33.3%)
**Level of training (Y1/Y2/Y3)***	4 (10.8%) / 16 (43.2%) / 17 (45.9%)	NA	NA	NA
**Years as professional** (median, range)**	NA	23.5 (7–33)	15 (5–43)	14 (7–17)
**N of focus groups/interviews**	5/6	3/0	2/1	2/0
**Total participants in focus groups (range in each focus group)**	31 (5-8)	18 (5-7)	5(2-3)	12 (6)

Table 2 - Demographic data.
* only applicable for trainees. **only applicable for supervisors, MR and NP

In short

The allocation of patients to either a GP trainee or GP supervisor, and therefore trainees' patient mix, is influenced by several interacting factors. The relationship between doctor (supervisor or trainee) and patient in the context of the patient's health problem played an essential role. Trainees obviously did not have a trustful relationship with patients yet, but both they and team members could compensate for this in various ways. Whether this occurs in practice depended on three influencing factors: trainees' behaviour, attitudes of the team, and context (Fig 1). Below we will first present how the patient-doctor relationship affects

For peer review only - http://bmjopen.bmj.com/site/about/guidelines.xhtml

trainees' patient mix. Subsequently, we will explain how practices may overcome the lack in trusting relationship between trainee and patient, and how this process is influenced by trainees' behaviour, attitudes of the team and the context (influencing factors).

[**Figure 1** - Factors influencing the patient mix of general practitioner trainees]

Disease characteristics & doctor–patient relationship

According to our participants, a central factor affecting the patient mix of trainees was the doctor–patient relationship in relation to the patient's health problem. The interviewees noticed that patients generally preferred to see their 'own' doctor, because the doctor knew the patient and his/her (disease) history and social context and was trusted. However, the patient's preference could change depending on the perceived severity of their health problems. For minor ailments or urgent problems patients were prepared to see the trainee because quick access or timeliness became more important.

NP no. 415: If someone comes with, for instance, heart rhythm disturbances or chest pain, they are happy if they can be seen quickly and that can also be a trainee. But if they come with mental health problems, and their GP knows the family situation etc, then they prefer to see that doctor, because he/she knows about them and their situation.

In general, our participants expressed it was felt that trainees were not in a practice long enough to really become the trusted doctor. This lack of a trainee–patient relationship seemed to affect the patient mix, especially when patients came with chronic and/or complex conditions.

For peer review only - http://bmjopen.bmj.com/site/about/guidelines.xhtml

Trainee no E1: Yes, I think that, this year, that I have had relatively little experience with chronic health problems and their treatment. If my supervisor is away a week, then it gets interesting. Otherwise they are mainly seen by my supervisor.

Overcoming the lack of doctor– patient relationship

Transference of trust

Findings showed that team members trusted by patients, such as the medical receptionist and the supervisor, could transfer this trust to the trainee, by emphasizing the trainee's benefits. For example trainees' previous work experience, pointing out that the trainee would have more time for them, and might have a new take on their problems. Medical receptionists in particular used these arguments if a patient was in doubt about who to see:

MR no 3: But sometimes if they've [the patient] had a problem for a long time, I'll say " Who knows, give it [red: seeing the trainee] a go, it may shed new light on the problem. And then they say "OK, I'll try it".

This process could be strengthened by the presence of the supervisor or nurse practitioner during the first consultation of the trainee with the patient. If all went well, the patient could be transferred to the care of the trainee:

Trainee no 2: I had a patient who needed palliative care in the first 2 weeks of the year and then my supervisor said to the patient], when we were at his bedside "Look, I trust [name of trainee], it's OK. I have to go to a conference; he'll take over". I hardly knew the patient but he said " OK, we'll do that" And everything went OK. But because he [red: the supervisor] made it explicit at that moment, it took away a lot of worry and stress.

For peer review only - http://bmjopen.bmj.com/site/about/guidelines.xhtml

Acquisition of trust

Although trainees obviously did not immediately have a trustful doctor–patient relationship, this could develop rapidly, often after a successful first encounter.

MR no 1: But if they [patients] have seen [a trainee] once, perhaps because their 'own' doctor couldn't see them, and that goes well, then there is no problem with subsequent appointments.

Whether the trainee gained the patient's trust and developed a good patient–doctor relationship depended to a large extent on the trainee's behaviour (see below).

Influencing factors

Trainees' behaviour

Trainees could affect their patient mix directly or indirectly. Directly, for example, if they invested time and energy in building a relationship with their patients, trainees could become the preferred doctor.

Trainee nr E2: I had a female patient with a BIRADS-4 [red: Breast Imaging Reporting and Data System (BIRADS). A standardized reporting system for mammography, BIRADS 4 indicates that a suspicious node has been seen] and I had to tell her. And that was population screening. She started treatment. I phoned her every 2 weeks. She said "I appreciate you phoning to ask how things are going". I try to do that sort of thing. Yes, I think you can gain people's trust in that way.

For peer review only

Trainees could influence their patient mix more indirectly by creating a supportive work environment. As stated earlier, trainees are dependent on other team members when it comes to patient allocation or transfer. However, these team members first need to feel that they can trust the trainee before they will allocate patients to the trainee. Trainees could influence this in several ways, one of which was being keen, as explained by the following medical receptionist who was asked what trainees can do to speed up trust:

MR3: Well, by mentioning it – if you have patients with this or that problem, let me see them. Some trainees do this. I can't think of anything specific at the moment, but if they say "I find that interesting, you can send them to me".

Trainees should also try to establish a good relationship with other staff, as explained below:

Supervisor no 17: What also may help, especially in the beginning of their training year, is that, if a trainee does not have a full surgery, that they do not stay in their room, but go to the medical receptionist. Personal contact between receptionists and trainees. The receptionist will work harder for the trainee, if they have a good relationship.

Trainees should also try to involve other staff in their training and learning, because this made staff more willing to allocate patients with a specific problem to them, as the following examples show:

MR no 2: Yes, If I know from this or that, hat she needs to discuss it at the educational day at the institute this week. I'll indeed try to plan it.

Attitude of team members

First encounters between patient and trainee are mainly arranged by supervisors, medical receptionists, and nurse practitioners. However, team members sometimes struggled with

For peer review only - http://bmjopen.bmj.com/site/about/guidelines.xhtml

achieving a balance between teaching the trainee and providing good patient care, which could affect the trainee patient mix. This is illustrated by the following conversation between two supervisors:

Supervisor no 2: I sometimes find it difficult. Recently, a woman phoned for me when I wasn't at the practice. She has recurrent lung cancer. The trainee took the call. Should I take over? should I contact the patient? Who should be in charge. I find it difficult.

Supervisor no 5:What do you find difficult?

Supervisor no 2:You don't want to let the patient go, you want the patient to know that you're there for her. On the other hand, she [the trainee] should also get the chance to do things.

The feeling of being involved in the trainee's education was important. As shown above, the nurse practitioner and medical receptionist were more willing to take the trainee's learning objectives into consideration when allocating a patient if they felt involved in that trainee's education. However, not every team member was equally involved or wanted to be involved.

Context

Our participants noticed that the context has also an important influence. For example, every practice has their own contextual factors such as the availability of a nurse practitioner, the organization and location of a practice, the size of a team, the number of previous trainees, and so on. An example of a contextual factor (having a walk-in surgery) that influenced trainee patient mix is explained by a supervisor:

Supervisor no 10: The walk-in surgery is always run by trainees in this practice. They can learn about minor acute problems but also make contact with patients, make an follow-up

For peer review only - http://bmjopen.bmj.com/site/about/guidelines.xhtml

appointment with them. In this way you can build up your own patient list in no time. It's very satisfactory and works well.

Context also seemed to affected strength of the doctor–patient relationship. If a general practice has only one doctor, then the doctor–patient relationship is probably strong and patients will be less willing to consult a trainee. This in contrast with larger practices where patients rotate between doctors, whereby patients have less strong ties with a single doctor.

Trainee no 15: I worked in a solo practice with a 64-year-old GP who had worked there for 34 years. He was THE doctor. So yes, it's probably good to gain experience in a health centre with eight GPs and three trainees, but whether it makes a difference to the patients? I don't know. In a solo practice the trusting relationship is very sure and resistant, certainly if the doctor has been there for long. Maybe somebody here has experience with this? To be in a health center with multiple doctors?

Trainee no 16: Yes, I think that you [the doctor] will be more replaceable. When a patient always sees the same GP, than he always wants to go there. If there are at least 6 doctors it [the patient] would be more flexible, so I think it matters.

For peer review only - http://bmjopen.bmj.com/site/about/guidelines.xhtml

For peer review only

Discussion

Summary of main findings

The mix of patients GP trainees see is shaped by a several factors. The characteristics of the health problem and the bond between trainee and patient are major determinants of this mix. Patients with complex problems tend to prefer their 'own doctor', because they trust him/her. It is generally recognized that a strong, trustful doctor–patient relationship is essential for the provision of high-quality care. Trainees cannot have such a relationship, especially not at the start of their training year in a new general practice. Our findings suggest that trainees and team members can take steps to surmount this lack of a trustful relationship. Team members can provide trainees with opportunities to see specific patients, and trainees can take specific steps to develop trustful doctor–patient relationships. Whether this happens depends on trainee behaviour, the attitudes of team members, and the context of the general practice. Team members have to trust the trainee before they are willing to allocate or transfer patients with complex medical conditions to the trainee. This is influenced by both practice culture and the trainee's behaviour. Trainees should invest in a good relationship with team members involve them in their development and needs, and show a keenness to learn. Trainees should also invest time and energy in building a relationship with patients, by being pro-active. This will help patients to trust them. In this way, trainees will generate their own list of patients with diverse medical problems of varying complexity. By doing so, they deliberately influence their own learning process in a positive way.

Strengths & Limitations

In this study we took a broad point of view and incorporate the perspectives from multiple participants with different roles within the general practice. We included four different

For peer review only - http://bmjopen.bmj.com/site/about/guidelines.xhtml

professional roles within the general practice (GP supervisor, GP trainee, medical receptionist and nurse practitioner). Because of this variety in roles, we had to include a relatively large number of participants before we reached data saturation. Although we did not incorporate patients' perspectives, findings from previous studies on patients' perspectives indicate that their views overlap with our(8, 28, 29). The use of qualitative methods enabled us to look in depth at factors affecting the patient mix of GP trainees from different perspectives. The fact that our findings are based on the patient mix of a Dutch GP trainee could be regarded as a limitation for translating our findings to other settings. However, the importance of continuity of care, especially for patients with chronic conditions, is acknowledged overall (28, 30-32). So this factor will probably influence the patients seen by GP trainees everywhere. However, it is less likely that our findings can be translated to a hospital setting, where the strong doctor–patient relationship seen in primary care is less likely to exist, because doctor–patient contact there is more fleeting.

Comparison with previous research and theory

A body of knowledge exists about the continuity of care, its relationship with the complexity of disease, and the high value patients and doctors place on it(29, 30, 33). However, only a few studies have investigated this in relation to the mix of patients seen by GP trainees, and these studies have tended to take the patients' point of view as outcome(8, 15, 29, 34).
Although we had a different perspective, our findings are in line with this research. Bonney et al. found that older patients have a strong preference for their 'own' GP, and that their attitude towards the GP trainee has to be viewed within this perspective(34). This finding is consistent with multiple cross-sectional studies from different countries(10, 28, 29, 35).
However, not only patients' preferences influenced the trainees' patient mix. We found that supervisors, nurse practitioners, and medical receptionists also have their influence. This

For peer review only - http://bmjopen.bmj.com/site/about/guidelines.xhtml

finding mirrors those of Bannister et al., who found that nurses and other team members in a hospital setting can hinder paediatric trainees in their learning opportunities(6). Our finding of trainees as important influencers is consistent with the findings of multiple studies on how trainees learn. Among others, previous research has found that learner engagement is important for trainees to practice skills(6), to create learning opportunities(36), and to create a more learning-oriented environment (37, 38).

Our findings are consistent with socio-cultural theories about learning(39). The situated learning theory of Lave and Wenger emphasizes the importance of participation in daily work to learning(40). Learners are active participants, and through participation they become full members of a 'community of practice'. Newcomers evolve through engagement with their workplace and gain more responsibilities. We found that GP trainees need to engage in their communities: by actively participating and building work relationships, trainees will gain trust and gradually be given more responsibility. Trainees evolve then from a 'legitimate peripheral participator' into a legitimate member of the community. We found that trainees who made an effort and invested time in their work environment were important influencers of their learning and patient mix. This finding is consistent with earlier work by Billet and Bandura, who stated that learners have to engage to reach the full educational potential a workplace has to offer(17, 41).

Implications for research and practice

The mix of patients seen by GP trainees should offer trainees the opportunities they need to develop competencies and to acquire skills. This study reveals that trainees' patient mix is affected by many entangled factors, but that trainees can take a central role in influencing

For peer review only - http://bmjopen.bmj.com/site/about/guidelines.xhtml

their patient mix, both direct and indirectly. These findings have some implications for practice

First, trainees should be aware that they can directly influence their own patient mix. Following our results, we recommend enlarging trainees' knowledge of the effects of the patient-doctor relationship and of ways in which they may speed up the bonding between themselves and their patients. For example, trainees can be taught to deliberately make social house visits, contact patients after their visit to the hospital, or arrange that all follow-up visits will be with the trainee. Second, trainees should be encouraged to arrange with their supervisor or the nurse practitioner to transfer some patients to the trainee's care. However, trainees and their supervisors should be informed that with this transfer, a *transfer of trust* must also take place. Seeing a patient together with the trainee and supervisors/nurse practitioner or stressing the trainee's qualities may help ensure that patients receive the same quality of care with the trainee as they do with their former doctor.

Furthermore, trainees and supervisors should be informed that relationships between trainees and the team members of a practice indirectly influence trainees' patient mix. Therefore, trainees should be instructed to maintain good relationships by showing keenness, involving other team members in their learning, conversing with the medical receptionist about their experiences during consultations. Moreover, supervisors and nurse practitioners should be aware that their own perceptions and beliefs about patient care influence the patients a trainee sees. We recommend starting the training year with a discussion between the trainee, supervisor, and other team members about these beliefs and the trainee's expectations.

The above mentioned recommendations of this study, together with the results a subsequent study on patients' perspectives, will be used to design an educational intervention to enhance

For peer review only - http://bmjopen.bmj.com/site/about/guidelines.xhtml

GP trainees patient mix. This intervention study will led to even more concrete and substantiated recommendations for trainees, supervisors and GP training program directors.

For peer review only - http://bmjopen.bmj.com/site/about/guidelines.xhtml

References

1. Teunissen PW, Scheele F, Scherpbier AJ, et al. How residents learn: qualitative evidence for the pivotal role of clinical activities. Med Educ. 2007;41(8):763-70.

2. Sagasser MH, Kramer AWM, Fluit C, et al. Self-entrustment: how trainees' self-regulated learning supports participation in the workplace. Adv Health Sci Educ Theory Pract. 2017;22(4):931-49.

3. Ericsson KA. Deliberate practice and the acquisition and maintenance of expert performance in medicine and related domains. Acad Med. 2004;79(10 Suppl):S70-81.

4. Ericsson KA. Acquisition and Maintenance of Medical Expertise: A Perspective From the Expert-Performance Approach With Deliberate Practice. Acad Med. 2015;90(11):1471-86.

5. Ten Cate O, Gruppen LD, Kogan JR, et al. Time-Variable Training in Medicine: Theoretical Considerations. Acad Med. 2018;93(3S Competency-Based, Time-Variable Education in the Health Professions):S6-S11.

6. Bannister SL, Dolson MS, Lingard L, et al. Not just trust: factors influencing learners' attempts to perform technical skills on real patients. Med Educ. 2018.

7. de Jong J, Visser MR, Wieringa-de Waard M. Steering the patient mix of GP trainees: results of a randomized controlled intervention. Med Teach. 2013;35(2):101-8.

8. Bonney A, Phillipson L, Reis S, et al. Patients' attitudes to general practice registrars: a review of the literature. Educ Prim Care. 2009;20(5):371-8.

9. Bonney A, Jones SC, Iverson D, et al. Trust, continuity and agency: keys to understanding older patients' attitudes to general practice trainees. Educ Health (Abingdon). 2014;27(1):39-46.

For peer review only

10. Nakar S, Levi D, Rosenberg R, et al. Patient attitudes to being treated by junior residents in the community. Patient Educ Couns. 2010;78(1):111-6.

11. Mol SS, Peelen JH, Kuyvenhoven MM. Patients' views on student participation in general practice consultations: a comprehensive review. Med Teach. 2011;33(7):e397-400.

12. Sagasser MH, Kramer AW, van der Vleuten CP. How do postgraduate GP trainees regulate their learning and what helps and hinders them? A qualitative study. BMC Med Educ. 2012;12:67.

13. van der Zwet J, Zwietering PJ, Teunissen PW, et al. Workplace learning from a socio-cultural perspective: creating developmental space during the general practice clerkship. Adv Health Sci Educ Theory Pract. 2011;16(3):359-73.

14. Sagasser MH, Kramer AW, van Weel C, et al. GP supervisors' experience in supporting self-regulated learning: a balancing act. Adv Health Sci Educ Theory Pract. 2015;20(3):727-44.

15. Hauer KE, Ten Cate O, Boscardin C, et al. Understanding trust as an essential element of trainee supervision and learning in the workplace. Adv Health Sci Educ Theory Pract. 2014;19(3):435-56.

16. Wearne S, Dornan T, Teunissen PW, et al. General practitioners as supervisors in postgraduate clinical education: an integrative review. Med Educ. 2012;46(12):1161-73.

17. Billett S. Learning through health care work: premises, contributions and practices. Med Educ. 2016;50(1):124-31.

18. Chen HC, Teherani A. Workplace affordances to increase learner engagement in the clinical workplace. Med Educ. 2015;49(12):1184-6.

19. Berkhout JJ, Helmich E, Teunissen PW, et al. Context matters when striving to promote active and lifelong learning in medical education. Med Educ. 2018;52(1):34-44.

For peer review only - http://bmjopen.bmj.com/site/about/guidelines.xhtml

20. Deketelaere A, Kelchtermans G, Struyf E, et al. Disentangling clinical learning experiences: an exploratory study on the dynamic tensions in internship. Med Educ. 2006;40(9):908-15.

21. Stok-Koch L, Bolhuis S, Koopmans R. Identifying factors that influence workplace learning in postgraduate medical education. Educ Health (Abingdon). 2007;20(1):8.

22. van de Wiel MW, Van den Bossche P, Janssen S, et al. Exploring deliberate practice in medicine: how do physicians learn in the workplace? Adv Health Sci Educ Theory Pract. 2011;16(1):81-95.

23. Hauer KE, Oza SK, Kogan JR, et al. How clinical supervisors develop trust in their trainees: a qualitative study. Med Educ. 2015;49(8):783-95.

24. Sterkenburg A, Barach P, Kalkman C, et al. When do supervising physicians decide to entrust residents with unsupervised tasks? Acad Med. 2010;85(9):1408-17.

25. Stalmeijer RE, McNaughton N, Van Mook WN. Using focus groups in medical education research: AMEE Guide No. 91. Med Teach. 2014;36(11):923-39.

26. Faber MJ, Burgers JS, Westert GP. A sustainable primary care system: lessons from the Netherlands. J Ambul Care Manage. 2012;35(3):174-81.

27. Braun V, Clarke V. Using thematic analysis in psychology. Qualitative Research in Psychology. 2006;3:77-101.

28. Malcolm CE, Wong KK, Elwood-Martin R. Patients' perceptions and experiences of family medicine residents in the office. Can Fam Physician. 2008;54(4):570-1, 1 e1-6.

29. Pearlman J, Moran SL, Van Driel M, et al. Continuity of care in general practice vocational training: prevalence, associations and implications for training. Education for Primay Care. 2016;27(2):27-36.

For peer review only - http://bmjopen.bmj.com/site/about/guidelines.xhtml

30. Haggerty JL, Roberge D, Freeman GK, et al. Experienced continuity of care when patients see multiple clinicians: a qualitative metasummary. Ann Fam Med. 2013;11(3):262-71.

31. Mainous AG, 3rd, Baker R, Love MM, et al. Continuity of care and trust in one's physician: evidence from primary care in the United States and the United Kingdom. Fam Med. 2001;33(1):22-7.

32. Merriel SW, Salisbury C, Metcalfe C, et al. Depth of the patient-doctor relationship and content of general practice consultations: cross-sectional study. Br J Gen Pract. 2015;65(637):e545-51.

33. Ridd MJ, Lewis G, Peters TJ, et al. Patient-doctor depth-of-relationship scale: development and validation. Ann Fam Med. 2011;9(6):538-45.

34. Bonney A, Phillipson L, Jones SC, et al. Older patients' attitudes to general practice registrars - A qualitative study. Aust Fam Physician. 2009;38(11):927-31.

35. Crawford GH, Gutman A, Kantor J, et al. Patients' attitudes toward resident participation in dermatology outpatient clinics. J Am Acad Dermatol. 2005;53(4):710-2.

36. Berkhout JJ, Helmich E, Teunissen PW, et al. Exploring the factors influencing clinical students' self-regulated learning. Med Educ. 2015;49(6):589-600.

37. Nothnagle M, Anandarajah G, Goldman RE, et al. Struggling to be self-directed: residents' paradoxical beliefs about learning. Acad Med. 2011;86(12):1539-44.

38. Goldman E, Plack M, Smith J, et al. Learning in a chaotic enviroment. Journal of Workplace Learning. 2009;21(7):555-74.

39. Mann K, Dornan T, Teunissen PW. Perspectives on learning. In: Dornan T, Mann KV, Scherpbier A, et al., editors. Medical Education: Theory and Practice: Elsevier; 2011. p. 17-38.

For peer review only - http://bmjopen.bmj.com/site/about/guidelines.xhtml

40. Lave J, Wenger E. Situated Learning: legitimate pheripheral participation. Cambridge UK: Canbridge University Press; 1991 1991.

41. Bandura A. Social cognitive theory: an agentic perspective. Annu Rev Psychol. 2001;52:1-26.

For peer review only

For peer review only - http://bmjopen.bmj.com/site/about/guidelines.xhtml

Acknowledgements

The authors wish to thank all participants who were willing to share their thoughts about trainees' patient mix, as well as the staff of the GP specialty training of the Amsterdam UMC (location AMC) who helped us with the organisation of the focus groups and interviews.

Funding statement

This work is funded by a grant from ZonMW (grant number: 839130001)

Conflict of interest

We declare no competing interest

Authors Statement

SdB, MV and NvD designed the study. SdB, SvR and MV performed the interviews and analysis with support from NvD and AK. All authors discussed the results. SdB wrote the manuscript with input from all authors

Data sharing

The data for this paper may be obtained from the authors upon request.

Figure legends

Table 3- Recruitment methods and number, place and duration of the focus groups or interviews
* For logistic reasons, one medical receptionist was interviewed separately.

Table 4 - Demographic data.
* only applicable for trainees. **only applicable for supervisors, MR and NP

Figure 2 - Factors influencing the patient mix of general practitioner trainees

For peer review only

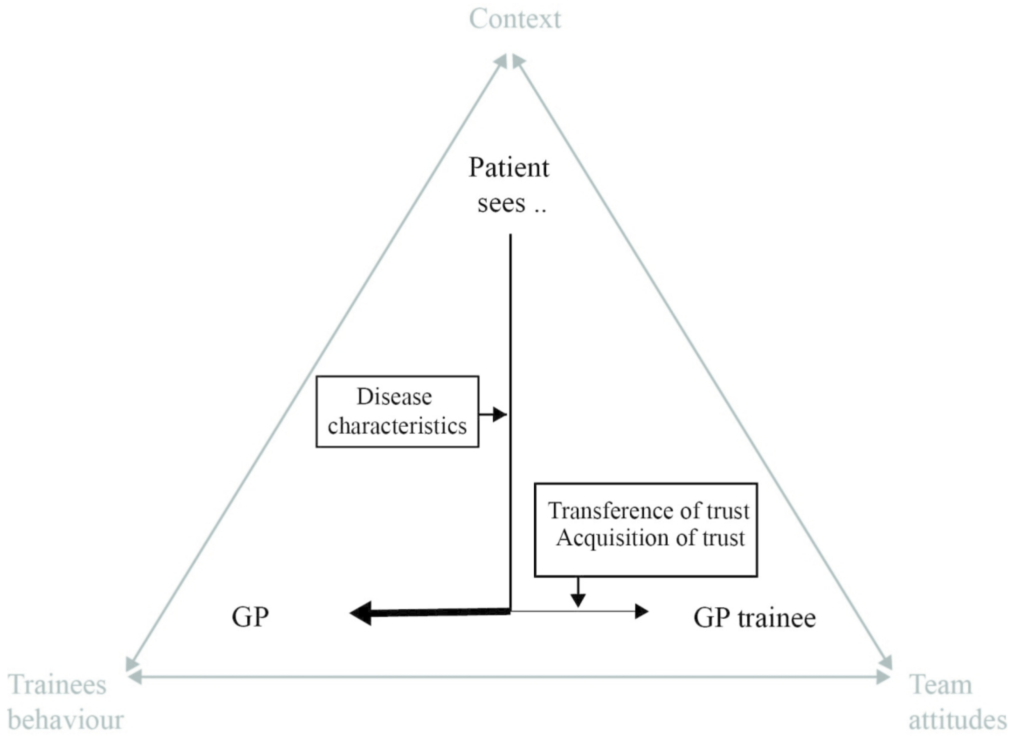

Figure 1 - Factors influencing the patient mix of general practitioner trainees

144x106mm (300 x 300 DPI)

Reporting checklist for qualitative study.

Based on the SRQR guidelines.

Instructions to authors

Complete this checklist by entering the page numbers from your manuscript where readers will find each of the items listed below.

Your article may not currently address all the items on the checklist. Please modify your text to include the missing information. If you are certain that an item does not apply, please write "n/a" and provide a short explanation.

Upload your completed checklist as an extra file when you submit to a journal.

In your methods section, say that you used the SRQRreporting guidelines, and cite them as:

O'Brien BC, Harris IB, Beckman TJ, Reed DA, Cook DA. Standards for reporting qualitative research: a synthesis of recommendations. Acad Med. 2014;89(9):1245-1251.

		Reporting Item	Page Number
**Title**			
	#1	Concise description of the nature and topic of the study identifying the study as qualitative or indicating the approach (e.g. ethnography, grounded theory) or data collection methods (e.g. interview, focus group) is recommended	1

For peer review only

Abstract

	#2	Summary of the key elements of the study using the abstract format of the intended publication; typically includes background, purpose, methods, results and conclusions	2

Introduction

Problem formulation	#3	Description and signifcance of the problem / phenomenon studied: review of relevant theory and empirical work; problem statement	5
Purpose or research question	#4	Purpose of the study and specific objectives or questions	6

Methods

Qualitative approach and research paradigm	#5	Qualitative approach (e.g. ethnography, grounded theory, case study, phenomenolgy, narrative research) and guiding theory if appropriate; identifying the research paradigm (e.g. postpositivist, constructivist / interpretivist) is also recommended; rationale. The rationale should briefly discuss the justification for choosing that theory, approach, method or technique rather than other options available; the assumptions and limitations implicit in those choices and how those choices influence study conclusions and transferability.	10

For peer review only

		As appropriate the rationale for several items might be discussed together.	
Researcher characteristics and reflexivity	#6	Researchers' characteristics that may influence the research, including personal attributes, qualifications / experience, relationship with participants, assumptions and / or presuppositions; potential or actual interaction between researchers' characteristics and the research questions, approach, methods, results and / or transferability	10
Context	#7	Setting / site and salient contextual factors; rationale	7
Sampling strategy	#8	How and why research participants, documents, or events were selected; criteria for deciding when no further sampling was necessary (e.g. sampling saturation); rationale	7-9
Ethical issues pertaining to human subjects	#9	Documentation of approval by an appropriate ethics review board and participant consent, or explanation for lack thereof; other confidentiality and data security issues	9
Data collection methods	#10	Types of data collected; details of data collection procedures including (as appropriate) start and stop dates of data collection and analysis, iterative process, triangulation of sources / methods, and modification of procedures in response to evolving study findings; rationale	8-10

For peer review only

Data collection instruments and technologies	#11	Description of instruments (e.g. interview guides, questionnaires) and devices (e.g. audio recorders) used for data collection; if / how the instruments(s) changed over the course of the study	9-10
Units of study	#12	Number and relevant characteristics of participants, documents, or events included in the study; level of participation (could be reported in results)	11
Data processing	#13	Methods for processing data prior to and during analysis, including transcription, data entry, data management and security, verification of data integrity, data coding, and anonymisation / deidentification of excerpts	10
Data analysis	#14	Process by which inferences, themes, etc. were identified and developed, including the researchers involved in data analysis; usually references a specific paradigm or approach; rationale	10
Techniques to enhance trustworthiness	#15	Techniques to enhance trustworthiness and credibility of data analysis (e.g. member checking, audit trail, triangulation); rationale	10

Results/findings

Syntheses and interpretation	#16	Main findings (e.g. interpretations, inferences, and themes); might include development of a theory or model, or integration with prior research or theory	11-12

For peer review only

Links to empirical data	#17	Evidence (e.g. quotes, field notes, text excerpts, photographs) to substantiate analytic findings	11-17
**Discussion**			
Intergration with prior work, implications, transferability and contribution(s) to the field	#18	Short summary of main findings; explanation of how findings and conclusions connect to, support, elaborate on, or challenge conclusions of earlier scholarship; discussion of scope of application / generalizability; identification of unique contributions(s) to scholarship in a discipline or field	18-20
Limitations	#19	Trustworthiness and limitations of findings	18-19
**Other**			
Conflicts of interest	#20	Potential sources of influence of perceived influence on study conduct and conclusions; how these were managed	26
Funding	#21	Sources of funding and other support; role of funders in data collection, interpretation and reporting	26

The SRQR checklist is distributed with permission of Wolters Kluwer © 2014 by the Association of American Medical Colleges. This checklist was completed on 06. June 2019 using https://www.goodreports.org/, a tool made by the EQUATOR Network in collaboration with Penelope.ai

For peer review only

BMJ Open

Professionals' perspectives on factors affecting GP trainees' patient mix: Results from an interview and focus group study among professionals working in Dutch general practice.

Journal:	*BMJ Open*
Manuscript ID	bmjopen-2019-032182.R2
Article Type:	Original research
Date Submitted by the Author:	11-Nov-2019
Complete List of Authors:	de Bever, Sarah; Amsterdam UMC - Locatie AMC, Department of General Practice/ GP Specialty Training Program<br>van Rhijn, Suzanne; Amsterdam UMC - Locatie AMC, Department of General Practice/ GP Specialty Training Program<br>van Dijk, Nynke; Amsterdam UMC - Locatie AMC, Department of General Practice/GP Specialty Training<br>Kramer, Anneke ; Leiden University Medical Center<br>Visser, Mechteld; Amsterdam UMC - Locatie AMC, Department of General Practice/GP Specialty Training
**Primary Subject Heading**:	Medical education and training
Secondary Subject Heading:	General practice / Family practice
Keywords:	MEDICAL EDUCATION & TRAINING, PRIMARY CARE, QUALITATIVE RESEARCH

SCHOLARONE™
Manuscripts

Professionals' perspectives on factors affecting GP trainees' patient mix: Results from an interview and focus group study among professionals working in Dutch general practice.

Authors: Sarah de Bever (MD)[a] , Suzanne C. van Rhijn (BSc)[a], Nynke van Dijk (MD, PhD)[a], Anneke W.M. Kramer (MD, PhD)[b], Mechteld R.M. Visser (PhD)[a]

Affliations:
a : Amsterdam UMC, University of Amsterdam, Department of General Practice/ GP Specialty Training Program, Amsterdam Public Health Research Institute, Amsterdam, Netherlands
b: Department of Public Health and Primary Care, Leiden University Medical Centre, Leiden, the Netherlands

Corresponding author: s.debever@amsterdamumc.nl. Adress: Meibergdreef 15, room J2-113, 1105 AZ Amsterdam, the Netherlands

Contact information:
Suzanne C. van Rhijn: suzannevanrhijn@hotmail.com
Nynke van Dijk: n.vandijk@amc.uva.nl
Anneke W.M. Kramer: a.w.m.kramer@lumc.nl
Mechteld. R.M. Visser: m.r.visser@amc.uva.nl

Word count: 4354 (excl abstract, strengths & limitations and references)

For peer review only

For peer review only - http://bmjopen.bmj.com/site/about/guidelines.xhtml

Abstract

Introduction: Seeing and treating patients in daily practice forms the basis of general practitioner (GP) training. However, the types of patients seen by GP trainees do not always match trainees' educational needs. Knowledge about factors that shape the mix of patient types is limited, especially with regard to the role of the professionals who work in the GP practice.

Aim: We investigated factors affecting the mix of patients seen by GP trainees from the perspective of professionals.

Design & Setting: This qualitative study involved GP trainees, GP supervisors, medical receptionists, and nurse practitioners affiliated with a GP Specialty Training Institute in the Netherlands.

Methods: Twelve focus groups and seven interviews with 73 participants were held. Data collection and analysis were iterative, using thematic analysis with a constant comparison methodology.

Results: The characteristics of patients' health problems and the bond between the doctor and patient are important determinants of GP trainees' patient mix. Because trainees have not yet developed bonds with patients, they are less likely to see patients with complex health problems. However, trainees can deliberately influence their patient mix by paying purposeful attention to bonding with patients and by gaining professional trust through focused engagement with their colleagues.

Conclusion: Trainees' patient mix is affected by various factors. Trainees and team members can take steps to ensure that this mix matches trainees' educational needs, but their success depends on the interaction between trainees' behavior, the attitudes of team members, and the

For peer review only - http://bmjopen.bmj.com/site/about/guidelines.xhtml

context. The findings show how the mix of patients seen by trainees can be influenced to become more trainee-centered and learning-oriented.

Keywords*: Postgraduate Medical Education, General Practice, Doctor-Patient Relationship. Workplace-Based Learning, Trust, Patient mix*

For peer review only

Strengths & limitations of the study

- We conducted focus groups and interviews with 73 participants who together represented all professional roles in general practice (GP, medical receptionist, nurse practitioner, GP trainee).
- Qualitative analysis allowed for an in-depth exploration of factors affecting trainees' patient mix.
- Combining all these perspectives, this study provides a rich insight into factors that affect trainees' patient mix.
- While patients' perspectives are missing from this study, literature on their perspectives suggest similar findings.
- The results are most applicable to settings in which continuity of care is important.

For peer review only
For peer review only - http://bmjopen.bmj.com/site/about/guidelines.xhtml

Introduction

Seeing and treating patients forms the basis of training for medical graduates specializing to become general practitioners (GPs)(1, 2). Thus their development of competence and skills depends on the mix of patients seen. According to Ericsson's theory of deliberate practice, mastery of competencies is achieved through repetitive practice, monitoring, and timely feedback from a trusted coach followed by further practice(3, 4). In the clinical workplace setting, this means repetitive cycles of the same patient symptoms or situations. Yet in everyday practice the mix of patients seen by GP trainees cannot always be organized in this way(5). The mix seems to be affected by a number of factors other than the educational needs of the trainee(6, 7). To make GP training more effective, a thorough understanding of all factors affecting the patient mix of GP trainees is needed.

Previous studies of trainees' patient mix mostly focused on patients' willingness to consult the trainee(8-11). However, trainees, their supervisors, and the practice environment probably also affect the patient mix. It has been found, for example, that GP trainees influence their patient mix in order to fill their knowledge gaps(12), but this requires developmental space(2, 13). Supervisors can provide this space, however they need to trust the trainee first(14-16). The relationship between GP trainee and supervisor affects the trainee's ability to steer his/her learning, and therefore probably also their patient mix(2, 12). Contextual factors, such as the work climate, may also influence which patients are seen by the trainee. While trainee workplaces should 'invite and support' learning(17-19), some are more work-oriented than supportive for learners(6, 20). For example, medical receptionists are responsible for allocating patients and they may not necessarily prioritize trainees' educational needs when allocating patients. Many studies have investigated how trainees learn(1, 12, 21, 22), how supervisors develop their trust(23, 24), the importance of work relationships, and the

For peer review only - http://bmjopen.bmj.com/site/about/guidelines.xhtml

influence of context on learning(2, 12, 19), but none have looked at the impact of the trainees' patient mix. Yet this information is needed in order to support training in the general practice. In this study, we investigated factors influencing the patient mix of GP trainees. To this end, we carried out a qualitative study involving different health professionals (GP trainees, their supervisors, medical receptionists, and nurse practitioners) in a general practice setting. Findings can be used to ensure that trainees see a mix of patients appropriate to their needs, thereby optimizing workplace-based learning.

For peer review only

For peer review only - http://bmjopen.bmj.com/site/about/guidelines.xhtml

Methods

Design

Focus group meetings and additional semi-structured interviews were used in this qualitative study. Focus groups were chosen because they provide the opportunity for in-depth investigation of a topic through group discussion(25).

Participants

GP trainees, GP supervisors, medical receptionists, and nurse practitioners affiliated with the GP Specialty Training Institute of the Amsterdam UMC (location AMC) were invited to participate. All participants came from different general practices. See Table 1 for more details regarding the number of participants; recruitment methods used; the number, place, and duration of the focus groups or interviews. After the initial analysis, we held six interviews with trainees to deepen our first impressions. We conducted purposive sampling on final year trainees, because they appeared to be the most informative participants. While all focus groups were homogenous with regard to profession we ensured that they were heterogeneous with regard to practice organization (single practice, group practice, or health center) and level of the trainee to stimulate discussion.

	**Design**	**Trainees (Y1/Y2/Y3)**	**Supervisors**	**Medical receptionist**	**Nurse practitioners**
**Recruitment**	Focus group	Multiple presentations during the educational program in 2016 & 2017	Email prior to training day June 2016 and presentation on training day April 2018	Presentation during training day March 2016	Email prior to training day April 2016

For peer review only - http://bmjopen.bmj.com/site/about/guidelines.xhtml

	Interview	By teachers of educational program	NA	Presentation during training day March 2016	NA
**Location/Time**	Focus group	Regular educational program day at the department between May and June 2016	Training day for supervisors in June 2016 (2) and April 2018 (1)	After work hours at GP department	Training day April 2016
	Interview	Trainees' place of preference between January and February 2017	NA	MRs' place of preference in December 2016*	NA
**Duration**	Focus group	40 minutes	60 minutes	60 minutes	60 minutes
	Interview	20 minutes	NA	30 minutes	NA

Table 1- Recruitment methods and number, place and duration of the focus groups or interviews
* For logistic reasons, one medical receptionist was interviewed separately.

Setting

In the Netherlands, all residents are registered with a general practice. GPs are generalists, gatekeepers for specialist and hospital care, and patients' first contact with the healthcare system (26). Most general practices are in the community, so there is often a strong, long-standing relationship between GPs and patients (26). Personal care and continuity of care are core values in Dutch general practice. GPs can work alone (solo practice), with two or more GPs (group practice), or be organized in larger health centers with several GPs and other

For peer review only - http://bmjopen.bmj.com/site/about/guidelines.xhtml

healthcare provides. In their practice, GPs are supported by one or more medical receptionists and nurse practitioners. In general, medical receptionists are responsible for allocating patients to specific doctors, while nurse practitioners are responsible for the care of patients with chronic diseases, elderly patients, or patients with psychosocial problems.

In the Netherlands, the three-year postgraduate GP training program is offered by eight departments of general practice affiliated with academic medical centers. In their first and third years, trainees work in a general practice four days each week. On the fifth day, trainees follow an educational program at one of the departments. In the general practice, each GP trainee is supervised and coached by an experienced GP (supervisor). Most of the time, trainees work independently but discuss their patients on a regular basis, or immediately if needed, with their designated supervisor. In their second year, trainees rotate through hospital, nursing home, and psychiatric care settings. GP supervisors, nurse practitioners, and medical receptionists affiliated with the GP Specialty Training Institute of the Amsterdam UMC, location AMC, are offered training days. Training days for GP supervisors are held multiple times a year and are compulsory. Training days for nurse practitioners and medical receptionists are held twice a year and are voluntary.

Patient and Public Involvement

We did not involve patients nor the public in this study. The research question was based on previous studies and was part of a larger, peer-reviewed study protocol. The results will be disseminated through training programs for GP supervisors and trainees. We developed workshops in which supervisors and trainees are informed about the main results of our study and are taught skills to influence their patient mix. These workshops are part of further

For peer review only - http://bmjopen.bmj.com/site/about/guidelines.xhtml

investigations on trainees' patient mix and are implemented within the GP specialty training program of the AMC.

Data collection

Topic lists, covering the role of trust, supervision, patients' perspectives and preferences, continuity of care, and contextual factors (for example, practice size and number of employees), were used for the focus meetings and interviews. Topic lists were continuously updated based on findings. Focus groups were led by skilled moderators experienced in qualitative research and familiar with the scope of this study. The first author (SdB) observed most of the focus groups. If this was not possible for logistic or ethical reasons, another skilled observer (SvR) familiar with the study protocol took over. All focus groups and interviews were audio-taped and transcribed verbatim. Transcripts were pseudonymized.

Data analysis

We used thematic analysis with a constant comparison methodology to guide our analysis(27). Initially two team members (SdB and SvR) independently coded two transcripts. After these first two transcripts, SdB and SvR discussed the initial coding scheme until they reached consensus. Next, five more transcripts were independently coded by SdB and SvR, using constant comparison with the earlier codes and adding new codes were needed. These codes were discussed in detail between SdB, SvR and MV until consensus was reached. Data from subsequent transcripts were further coded by SdB using the discussed codes scheme and, where needed, new codes were added and other code were merged or adopted. During this phase SdB, SvR and MR met on regular basis to discuss the code scheme and overarching themes. To fully explore the themes and their mutual relationship, multiple diagrams and figures were created. After proximally 13 transcripts the code scheme seemed sufficient to code the last transcripts, indicating data saturation. This final code scheme and the earlier

For peer review only - http://bmjopen.bmj.com/site/about/guidelines.xhtml

figures were discussed within the whole research group. The research team included a GP trainee/PhD student (SdB) and four researchers, all experienced in medical education research and qualitative methodology: an educationalist and medical doctor (NvD), a cognitive psychologist (MV), a general practitioner (AK), and a research assistant/educationalist (SvR).

Ethics approval

Prior to each focus group or interview, participants were given study information and informed consent was obtained. The study was approved by the ethics review board of the Dutch Association for Medical Education (file number 630).

For peer review only

Results

In total, eleven focus groups were held, of which five with GP trainees (one with first-year trainees, two with second-year trainees, two with third-year trainees), three with supervisors, two with nurse practitioners, and two with medical receptionists. Additionally, six interviews were held with third-year trainees before data saturation was reached. In total, we included 73 participants. The participants' demographics can be found in Table 2.

	Trainees (n=37)	Supervisors (n=18)	Medical receptionists (n=6)	Nurse practitioners (n=12)
**Age (median, range)**	30 (27-35)	56 (35-62)	53 (39-60)	53 (38-63)
**Gender (M/F)**	6 (16.2%) /31 (83.8%)	5 (27.8%) / 13 (72.2%)	0/6 (100%)	0/12 (100%)
**Practice organization**				
• **Single**	12 (32.4%)	5 (27.8%)	0	1 (8.3%)
• **Group**	16 (43.2%)	10 (55.6%)	5 (83.3%)	7 (58.3%)
• **Health care centre**	3 (21.6%)	3 (16.7%)	1 (16.6%)	4 (33.3%)
**Level of training (Y1/Y2/Y3)***	4 (10.8%) / 16 (43.2%) / 17 (45.9%)	NA	NA	NA
**Years as professional** (median, range)**	NA	23.5 (7–33)	15 (5–43)	14 (7–17)
**N of focus groups/interviews**	5/6	3/0	2/1	2/0
**Total participants in focus groups (range in each focus group)**	31 (5-8)	18 (5-7)	5(2-3)	12 (6)

Table 2 - Demographic data.
* only applicable for trainees. **only applicable for supervisors, MR and NP

Our analysis revealed three major themes that together explain which factors influence GP trainees' patient mix: *Disease characteristics & patient – doctor relationship*, *Overcoming the lack of patient – doctor relationship and Influencing factors*. Appendix I provides an overview and summary of each theme and their subthemes.

The emerged themes are closely interrelated with each other. For a thorough understanding of our findings, an understanding of these relationship is needed. Therefore, we will first start with a summary of our findings and how they relate to each other, before explaining the themes and their subthemes separately.

For peer review only - http://bmjopen.bmj.com/site/about/guidelines.xhtml

In short

The allocation of patients to either a GP trainee or GP supervisor, and therefore trainees' patient mix, is influenced by several interacting factors. The relationship between doctor (supervisor or trainee) and patient in the context of the patient's health problem played an essential role. Trainees obviously did not have a trustful relationship with patients yet, but both they and team members could compensate for this in various ways. Whether this occurs in practice depended on three influencing factors: trainees' behaviour, attitudes of the team, and context (Fig 1). Below we will first present how the patient-doctor relationship affects trainees' patient mix. Subsequently, we will explain how practices may overcome the lack in trusting relationship between trainee and patient, and how this process is influenced by trainees' behaviour, attitudes of the team and the context (influencing factors).

[**Figure 1** - Factors influencing the patient mix of general practitioner trainees]

Disease characteristics & doctor–patient relationship

According to our participants, a central factor affecting the patient mix of trainees was the doctor–patient relationship in relation to the patient's health problem. The interviewees noticed that patients generally preferred to see their 'own' doctor, because the doctor knew the patient and his/her (disease) history, social context and was trusted. However, patient's preference could change depending on the perceived severity of their health problems.

NP no. 415: If someone comes with, for instance, heart rhythm disturbances or chest pain, they are happy if they can be seen quickly and that can also be a trainee. But if they come with mental health problems, and their GP knows the family situation etc, then they prefer to see that doctor, because he/she knows about them and their situation.

For peer review only - http://bmjopen.bmj.com/site/about/guidelines.xhtml

In general, our participants expressed it was felt that trainees were not long enough in a practice to really become the trusted doctor. This lack of a trainee–patient relationship seemed to affect the patient mix, especially when patients came with chronic and/or complex conditions.

Trainee no E1: Yes, I think that, this year, that I have had relatively little experience with chronic health problems and their treatment. If my supervisor is away a week, then it gets interesting. Otherwise they are mainly seen by my supervisor.

Overcoming the lack of doctor– patient relationship

Transference of trust

Findings showed that team members trusted by patients, such as the medical receptionist and the supervisor, could transfer this trust to the trainee by emphasizing the trainee's benefits. For example, trainees' previous work experience, pointing out that the trainee would have more time for them, and might have a new take on their problems. Medical receptionists in particular used these arguments if a patient was in doubt about who to see:

MR no 3: But sometimes if they've [the patient] had a problem for a long time, I'll say " Who knows, give it [red: seeing the trainee] a go, it may shed new light on the problem. And then they say "OK, I'll try it".

This process could be strengthened by the presence of the supervisor or nurse practitioner during the first consultation of the trainee with the patient. If all went well, the patient could be transferred to the care of the trainee:

For peer review only - http://bmjopen.bmj.com/site/about/guidelines.xhtml

Trainee no 2: I had a patient who needed palliative care in the first 2 weeks of the year and then my supervisor said to the patient], when we were at his bedside "Look, I trust [name of trainee], it's OK. I have to go to a conference; he'll take over". I hardly knew the patient but he said " OK, we'll do that" And everything went OK. But because he [red: the supervisor] made it explicit at that moment, it took away a lot of worry and stress.

Acquisition of trust

Although trainees obviously did not immediately have a trustful doctor–patient relationship, this could develop rapidly, often after a successful first encounter.

MR no 1: But if they [patients] have seen [a trainee] once, perhaps because their 'own' doctor couldn't see them, and that goes well, then there is no problem with subsequent appointments.

Whether the trainee gained the patient's trust and developed a good patient–doctor relationship depended to a large extent on the trainee's behaviour (see below).

Influencing factors

Trainees' behaviour

Trainees could affect their patient mix directly or indirectly. Directly through investing time and energy in building a relationship with their patients, through which trainees could become the preferred doctor.

For peer review only - http://bmjopen.bmj.com/site/about/guidelines.xhtml

Trainee nr E2: I had a female patient with a BIRADS-4 [red: Breast Imaging Reporting and Data System (BIRADS). A standardized reporting system for mammography, BIRADS 4 indicates that a suspicious node has been seen] and I had to tell her. And that was population screening. She started treatment. I phoned her every 2 weeks. She said "I appreciate you phoning to ask how things are going". I try to do that sort of thing. Yes, I think you can gain people's trust in that way.

Trainees could influence their patient mix more indirectly by creating a supportive work environment. As stated earlier, trainees are dependent on other team members when it comes to patient allocation or transfer. However, these team members first need to feel that they can trust the trainee before they will allocate patients to the trainee. Trainees could influence this in several ways, one of which was being keen, as explained by the following medical receptionist who was asked what trainees can do to speed up trust:

MR3: Well, by mentioning it – if you have patients with this or that problem, let me see them. Some trainees do this. I can't think of anything specific at the moment, but if they say "I find that interesting, you can send them to me".

Trainees should also try to establish a good relationship with other staff, as explained below:

Supervisor no 17: What also may help, especially in the beginning of their training year, is that, if a trainee does not have a full surgery, that they do not stay in their room, but go to the medical receptionist. Personal contact between receptionists and trainees. The receptionist will work harder for the trainee, if they have a good relationship.

For peer review only

Trainees should also try to involve other staff in their training and learning, because this made staff more willing to allocate patients with a specific problem to them, as the following examples show:

MR no 2: Yes, If I know from this or that, hat she needs to discuss it at the educational day at the institute this week. I'll indeed try to plan it.

Attitude of team members

First encounters between patient and trainee are mainly arranged by supervisors, medical receptionists, and nurse practitioners. However, team members sometimes struggled with achieving a balance between teaching the trainee and providing good patient care, which could affect the trainee patient mix. This is illustrated by the following conversation between two supervisors:

Supervisor no 2: I sometimes find it difficult. Recently, a woman phoned for me when I wasn't at the practice. She has recurrent lung cancer. The trainee took the call. Should I take over? should I contact the patient? Who should be in charge. I find it difficult.

Supervisor no 5:What do you find difficult?

Supervisor no 2:You don't want to let the patient go, you want the patient to know that you're there for her. On the other hand, she [the trainee] should also get the chance to do things.

The feeling of being involved in the trainee's education was important. As shown above, the nurse practitioner and medical receptionist were more willing to take the trainee's learning objectives into consideration when allocating a patient if they felt involved in that trainee's education. However, not every team member was equally involved or wanted to be involved.

For peer review only - http://bmjopen.bmj.com/site/about/guidelines.xhtml

Context

Our participants noticed that the context has also an important influence. For example, every practice has their own contextual factors such as the availability of a nurse practitioner, the organization and location of a practice, the size of a team, the number of previous trainees, and so on. An example of a contextual factor (having a walk-in surgery) that influenced trainee patient mix is explained by a supervisor:

Supervisor no 10: The walk-in surgery is always run by trainees in this practice. They can learn about minor acute problems but also make contact with patients, make an follow-up appointment with them. In this way you can build up your own patient list in no time. It's very satisfactory and works well.

Context also seemed to affected strength of the doctor–patient relationship. If a general practice has only one doctor, then the doctor–patient relationship is probably strong and patients will be less willing to consult a trainee. This in contrast with larger practices where patients rotate between doctors, whereby patients have less strong ties with a single doctor.

Trainee no 15: I worked in a solo practice with a 64-year-old GP who had worked there for 34 years. He was THE doctor. So yes, it's probably good to gain experience in a health centre with eight GPs and three trainees, but whether it makes a difference to the patients? I don't know. In a solo practice the trusting relationship is very sure and resistant, certainly if the doctor has been there for long. Maybe somebody here has experience with this? To be in a health center with multiple doctors?

For peer review only - http://bmjopen.bmj.com/site/about/guidelines.xhtml

Trainee no 16: Yes, I think that you [the doctor] will be more replaceable. When a patient always sees the same GP, than he always wants to go there. If there are at least 6 doctors it [the patient] would be more flexible, so I think it matters.

For peer review only

For peer review only - http://bmjopen.bmj.com/site/about/guidelines.xhtml

Discussion

Summary of main findings

The mix of patients GP trainees see is shaped by a several factors. The characteristics of the health problem and the bond between trainee and patient are major determinants of this mix. Patients with complex problems tend to prefer their 'own doctor', because they trust him/her. It is generally recognized that a strong, trustful doctor–patient relationship is essential for the provision of high-quality care. Trainees cannot have such a relationship, especially not at the start of their training year in a new general practice. Our findings suggest that trainees and team members can take steps to surmount this lack of a trustful relationship. Team members can provide trainees with opportunities to see specific patients, and trainees can take specific steps to develop trustful doctor–patient relationships. Whether this happens depends on trainee behaviour, the attitudes of team members, and the context of the general practice. Team members have to trust the trainee before they are willing to allocate or transfer patients with complex medical conditions to the trainee. This is influenced by both practice culture and the trainee's behaviour. Trainees should invest in a good relationship with team members, involve them in their development and needs, and show a keenness to learn. Trainees should also invest time and energy in building a relationship with patients, by being pro-active. This will help patients to trust them. In this way, trainees will generate their own list of patients with diverse medical problems of varying complexity and deliberately influence their own learning process in a positive way.

Strengths & Limitations

In this study we took a broad point of view and incorporate the perspectives from multiple participants with different roles within the general practice, namely, the GP supervisor, the

For peer review only - http://bmjopen.bmj.com/site/about/guidelines.xhtml

GP trainee, the medical receptionist and the nurse practitioner. Because of this variety in roles, we had to include a relatively large number of participants before we reached data saturation. Although we did not incorporate patients' perspectives, findings from previous studies on patients' perspectives indicate that their views overlap with our findings(8, 28, 29). The use of qualitative methods enabled us to look in depth at factors affecting the patient mix of GP trainees from different perspectives. The fact that our findings are based on the patient mix of a Dutch GP trainee could be regarded as a limitation for translating our findings to other settings. Though the importance of continuity of care, especially for patients with chronic conditions, is acknowledged overall (28, 30-32), contextual differences could affect the transferability of the influence this has on trainees patient mix elsewhere. For example, Dutch GPs are rooted within communities which strengthens the bond between patients and doctors. In other places, doctors can have less or more bonding with their community and therefore, this might have less or more influence on GP trainees' patient mix. Moreover, it is less likely that our findings can be translated to a hospital setting, where the strong doctor–patient relationship seen in primary care is less likely to exist, because doctor–patient contact there is more fleeting.

Comparison with previous research and theory

A body of knowledge exists about the continuity of care, its relationship with the complexity of disease, and the high value patients and doctors place on it(29, 30, 33). However, only a few studies have investigated this in relation to the mix of patients seen by GP trainees, and these studies have tended to take the patients' point of view as outcome(8, 15, 29, 34). Although we had a different perspective, our findings are in line with this research. Bonney et al. found that older patients have a strong preference for their 'own' GP, and that their attitude

For peer review only - http://bmjopen.bmj.com/site/about/guidelines.xhtml

towards the GP trainee has to be viewed within this perspective(34). This finding is consistent with multiple cross-sectional studies from different countries(10, 28, 29, 35).

However, not only patients' preferences influenced the trainees' patient mix. We found that supervisors, nurse practitioners, and medical receptionists also have their influence. This finding mirrors those of Bannister et al., who found that nurses and other team members in a hospital setting can hinder paediatric trainees in their learning opportunities(6). Our finding of trainees as important influencers is consistent with the findings of multiple studies on how trainees learn. Among others, previous research has found that learner engagement is important for trainees to practice skills(6), to create learning opportunities(36), and to create a more learning-oriented environment (37, 38).

Our findings are consistent with socio-cultural theories about learning(39). The situated learning theory of Lave and Wenger emphasizes the importance of participation in daily work for learning(40). Learners are active participants, and through participation they become full members of a 'community of practice'. Newcomers evolve through engagement with their workplace and gain more responsibilities. We found that GP trainees need to engage in their communities: by actively participating and building work relationships trainees will gain trust and gradually be given more responsibility. Trainees evolve then from a 'legitimate peripheral participator' into a legitimate member of the community. We found that trainees who made an effort and invested time in their work environment were important influencers of their learning and patient mix. This finding is consistent with earlier work by Billet and Bandura, who stated that learners have to engage to reach the full educational potential a workplace has to offer(17, 41).

For peer review only

Implications for research and practice

The mix of patients seen by GP trainees should offer trainees the opportunities they need to develop competencies and to acquire skills. This study reveals that trainees' patient mix is affected by many entangled factors, but that trainees can take a central role in influencing their patient mix, both direct and indirectly. These findings have some implications for practice.

First, trainees should be aware that they can directly influence their own patient mix. Following our results, we recommend enlarging trainees' knowledge of the effects of the patient-doctor relationship and of ways in which they may speed up the bonding between themselves and their patients. For example, trainees can be taught to deliberately make social house visits, contact patients after their visit to the hospital, or arrange that all follow-up visits will be with the trainee. Second, trainees should be encouraged to arrange with their supervisor or the nurse practitioner to transfer some patients to the trainee's care. However, trainees and their supervisors should be informed that with this transfer, a *transfer of trust* must also take place. Seeing a patient together with the trainee and supervisors/nurse practitioner or stressing the trainee's qualities may help ensure that patients receive the same quality of care with the trainee as they do with their former doctor.

Furthermore, trainees and supervisors should be informed that relationships between trainees and the team members of a practice indirectly influence trainees' patient mix. Therefore, trainees should be instructed to maintain good relationships by showing keenness, involving other team members in their learning, conversing with the medical receptionist about their experiences during consultations. Moreover, supervisors and nurse practitioners should be aware that their own perceptions and beliefs about patient care influence the patients a trainee

For peer review only - http://bmjopen.bmj.com/site/about/guidelines.xhtml

sees. We recommend starting the training year with a discussion between the trainee, supervisor, and other team members about these beliefs and the trainee's expectations.

The above mentioned recommendations of this study, together with the results a subsequent study on patients' perspectives, will be used to design an educational intervention to enhance GP trainees patient mix. This intervention study will led to even more concrete and substantiated recommendations for trainees, supervisors and GP training program directors.

For peer review only

For peer review only - http://bmjopen.bmj.com/site/about/guidelines.xhtml

References

1. Teunissen PW, Scheele F, Scherpbier AJ, et al. How residents learn: qualitative evidence for the pivotal role of clinical activities. Med Educ. 2007;41(8):763-70.

2. Sagasser MH, Kramer AWM, Fluit C, et al. Self-entrustment: how trainees' self-regulated learning supports participation in the workplace. Adv Health Sci Educ Theory Pract. 2017;22(4):931-49.

3. Ericsson KA. Deliberate practice and the acquisition and maintenance of expert performance in medicine and related domains. Acad Med. 2004;79(10 Suppl):S70-81.

4. Ericsson KA. Acquisition and Maintenance of Medical Expertise: A Perspective From the Expert-Performance Approach With Deliberate Practice. Acad Med. 2015;90(11):1471-86.

5. Ten Cate O, Gruppen LD, Kogan JR, et al. Time-Variable Training in Medicine: Theoretical Considerations. Acad Med. 2018;93(3S Competency-Based, Time-Variable Education in the Health Professions):S6-S11.

6. Bannister SL, Dolson MS, Lingard L, et al. Not just trust: factors influencing learners' attempts to perform technical skills on real patients. Med Educ. 2018.

7. de Jong J, Visser MR, Wieringa-de Waard M. Steering the patient mix of GP trainees: results of a randomized controlled intervention. Med Teach. 2013;35(2):101-8.

8. Bonney A, Phillipson L, Reis S, et al. Patients' attitudes to general practice registrars: a review of the literature. Educ Prim Care. 2009;20(5):371-8.

9. Bonney A, Jones SC, Iverson D, et al. Trust, continuity and agency: keys to understanding older patients' attitudes to general practice trainees. Educ Health (Abingdon). 2014;27(1):39-46.

For peer review only

10. Nakar S, Levi D, Rosenberg R, et al. Patient attitudes to being treated by junior residents in the community. Patient Educ Couns. 2010;78(1):111-6.

11. Mol SS, Peelen JH, Kuyvenhoven MM. Patients' views on student participation in general practice consultations: a comprehensive review. Med Teach. 2011;33(7):e397-400.

12. Sagasser MH, Kramer AW, van der Vleuten CP. How do postgraduate GP trainees regulate their learning and what helps and hinders them? A qualitative study. BMC Med Educ. 2012;12:67.

13. van der Zwet J, Zwietering PJ, Teunissen PW, et al. Workplace learning from a socio-cultural perspective: creating developmental space during the general practice clerkship. Adv Health Sci Educ Theory Pract. 2011;16(3):359-73.

14. Sagasser MH, Kramer AW, van Weel C, et al. GP supervisors' experience in supporting self-regulated learning: a balancing act. Adv Health Sci Educ Theory Pract. 2015;20(3):727-44.

15. Hauer KE, Ten Cate O, Boscardin C, et al. Understanding trust as an essential element of trainee supervision and learning in the workplace. Adv Health Sci Educ Theory Pract. 2014;19(3):435-56.

16. Wearne S, Dornan T, Teunissen PW, et al. General practitioners as supervisors in postgraduate clinical education: an integrative review. Med Educ. 2012;46(12):1161-73.

17. Billett S. Learning through health care work: premises, contributions and practices. Med Educ. 2016;50(1):124-31.

18. Chen HC, Teherani A. Workplace affordances to increase learner engagement in the clinical workplace. Med Educ. 2015;49(12):1184-6.

19. Berkhout JJ, Helmich E, Teunissen PW, et al. Context matters when striving to promote active and lifelong learning in medical education. Med Educ. 2018;52(1):34-44.

For peer review only

For peer review only - http://bmjopen.bmj.com/site/about/guidelines.xhtml

20. Deketelaere A, Kelchtermans G, Struyf E, et al. Disentangling clinical learning experiences: an exploratory study on the dynamic tensions in internship. Med Educ. 2006;40(9):908-15.

21. Stok-Koch L, Bolhuis S, Koopmans R. Identifying factors that influence workplace learning in postgraduate medical education. Educ Health (Abingdon). 2007;20(1):8.

22. van de Wiel MW, Van den Bossche P, Janssen S, et al. Exploring deliberate practice in medicine: how do physicians learn in the workplace? Adv Health Sci Educ Theory Pract. 2011;16(1):81-95.

23. Hauer KE, Oza SK, Kogan JR, et al. How clinical supervisors develop trust in their trainees: a qualitative study. Med Educ. 2015;49(8):783-95.

24. Sterkenburg A, Barach P, Kalkman C, et al. When do supervising physicians decide to entrust residents with unsupervised tasks? Acad Med. 2010;85(9):1408-17.

25. Stalmeijer RE, McNaughton N, Van Mook WN. Using focus groups in medical education research: AMEE Guide No. 91. Med Teach. 2014;36(11):923-39.

26. Faber MJ, Burgers JS, Westert GP. A sustainable primary care system: lessons from the Netherlands. J Ambul Care Manage. 2012;35(3):174-81.

27. Braun V, Clarke V. Using thematic analysis in psychology. Qualitative Research in Psychology. 2006;3:77-101.

28. Malcolm CE, Wong KK, Elwood-Martin R. Patients' perceptions and experiences of family medicine residents in the office. Can Fam Physician. 2008;54(4):570-1, 1 e1-6.

29. Pearlman J, Moran SL, Van Driel M, et al. Continuity of care in general practice vocational training: prevalence, associations and implications for training. Education for Primay Care. 2016;27(2):27-36.

For peer review only - http://bmjopen.bmj.com/site/about/guidelines.xhtml

30. Haggerty JL, Roberge D, Freeman GK, et al. Experienced continuity of care when patients see multiple clinicians: a qualitative metasummary. Ann Fam Med. 2013;11(3):262-71.

31. Mainous AG, 3rd, Baker R, Love MM, et al. Continuity of care and trust in one's physician: evidence from primary care in the United States and the United Kingdom. Fam Med. 2001;33(1):22-7.

32. Merriel SW, Salisbury C, Metcalfe C, et al. Depth of the patient-doctor relationship and content of general practice consultations: cross-sectional study. Br J Gen Pract. 2015;65(637):e545-51.

33. Ridd MJ, Lewis G, Peters TJ, et al. Patient-doctor depth-of-relationship scale: development and validation. Ann Fam Med. 2011;9(6):538-45.

34. Bonney A, Phillipson L, Jones SC, et al. Older patients' attitudes to general practice registrars - A qualitative study. Aust Fam Physician. 2009;38(11):927-31.

35. Crawford GH, Gutman A, Kantor J, et al. Patients' attitudes toward resident participation in dermatology outpatient clinics. J Am Acad Dermatol. 2005;53(4):710-2.

36. Berkhout JJ, Helmich E, Teunissen PW, et al. Exploring the factors influencing clinical students' self-regulated learning. Med Educ. 2015;49(6):589-600.

37. Nothnagle M, Anandarajah G, Goldman RE, et al. Struggling to be self-directed: residents' paradoxical beliefs about learning. Acad Med. 2011;86(12):1539-44.

38. Goldman E, Plack M, Smith J, et al. Learning in a chaotic enviroment. Journal of Workplace Learning. 2009;21(7):555-74.

39. Mann K, Dornan T, Teunissen PW. Perspectives on learning. In: Dornan T, Mann KV, Scherpbier A, et al., editors. Medical Education: Theory and Practice: Elsevier; 2011. p. 17-38.

For peer review only

40. Lave J, Wenger E. Situated Learning: legitimate pheripheral participation. Cambridge UK: Canbridge University Press; 1991 1991.

41. Bandura A. Social cognitive theory: an agentic perspective. Annu Rev Psychol. 2001;52:1-26.

For peer review only

For peer review only - http://bmjopen.bmj.com/site/about/guidelines.xhtml

Acknowledgements

The authors wish to thank all participants who were willing to share their thoughts about trainees' patient mix, as well as the staff of the GP specialty training of the Amsterdam UMC (location AMC) who helped us with the organisation of the focus groups and interviews.

Funding statement

This work is funded by a grant from ZonMW (grant number: 839130001)

Conflict of interest

We declare no competing interest

Authors Statement

SdB, MV and NvD designed the study. SdB, SvR and MV performed the interviews and analysis with support from NvD and AK. All authors discussed the results. SdB wrote the manuscript with input from all authors

Data sharing

The data for this paper may be obtained from the authors upon request.

Table 1- Recruitment methods and number, place and duration of the focus groups or interviews
* For logistic reasons, one medical receptionist was interviewed separately.

Table 2 - Demographic data.
* only applicable for trainees. **only applicable for supervisors, MR and NP

Figure 1 - Factors influencing the patient mix of general practitioner trainees

For peer review only

For peer review only - http://bmjopen.bmj.com/site/about/guidelines.xhtml

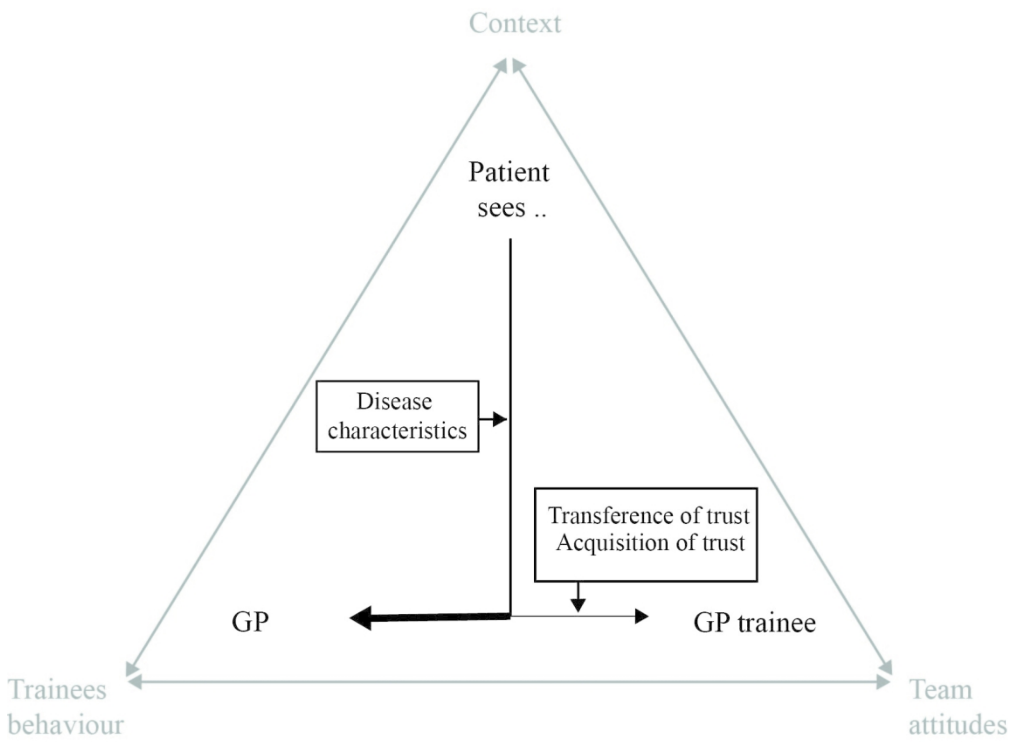

Figure 1 - Factors influencing the patient mix of general practitioner trainees

144x106mm (300 x 300 DPI)

Appendix I

Theme	Subtheme	Summary
Disease characteristics & patient – doctor relationship		Patients can have a strong preference for which doctor they want to consult. This preference is dependent on their complaint. Patients prefer to consult a trusted doctor when they have a complex complaint, and for minor ailments patients have less preference.
Overcoming lack in patient – doctor relationship		The time that trainees spend in a practice is relatively short for establishing a trusted relationship with patients. However, this lack in bond between trainee and patient can be surmounted.
	Transference of trust	Team members, who are trusted by patients, can 'transfer' this trust to the trainee. By doing so, they enhance bonding between trainee and patient.
	Acquisition of trust	Through good initial contact between trainees and patients, trainees can form a bond with patients.
Influencing factors		Whether or not a bond forms between trainees and patients depend on three factors
	Trainees behaviour	Trainees can directly and indirectly influence their patient mix. They can do this directly through bonding with patient by being an understanding, empathic doctor. Indirectly, trainees can influence their patient mix by influencing the willingness and trust team members have in them.
	Attitudes of team members	Team members can influence trainees' patient mix deliberately. However, whether or not they are willing to do so, depends on the relationship they have with the trainee, the trust they have in trainees' capabilities and the involvement in the education of the trainee.
	Context	The context of a training practice can hinder or help trainees' patient mix. It can also affects the strength in bond between doctors and patients.

Table 5 - Summary of the major themes and their subthemes

For peer review only

Reporting checklist for qualitative study.

Based on the SRQR guidelines.

Instructions to authors

Complete this checklist by entering the page numbers from your manuscript where readers will find each of the items listed below.

Your article may not currently address all the items on the checklist. Please modify your text to include the missing information. If you are certain that an item does not apply, please write "n/a" and provide a short explanation.

Upload your completed checklist as an extra file when you submit to a journal.

In your methods section, say that you used the SRQRreporting guidelines, and cite them as:

O'Brien BC, Harris IB, Beckman TJ, Reed DA, Cook DA. Standards for reporting qualitative research: a synthesis of recommendations. Acad Med. 2014;89(9):1245-1251.

		Reporting Item	Page Number
**Title**			
	#1	Concise description of the nature and topic of the study identifying the study as qualitative or indicating the approach (e.g. ethnography, grounded theory) or data collection methods (e.g. interview, focus group) is recommended	1

For peer review only

Abstract

	#2	Summary of the key elements of the study using the abstract format of the intended publication; typically includes background, purpose, methods, results and conclusions	2

Introduction

Problem formulation	#3	Description and signifcance of the problem / phenomenon studied: review of relevant theory and empirical work; problem statement	5
Purpose or research question	#4	Purpose of the study and specific objectives or questions	6

Methods

Qualitative approach and research paradigm	#5	Qualitative approach (e.g. ethnography, grounded theory, case study, phenomenolgy, narrative research) and guiding theory if appropriate; identifying the research paradigm (e.g. postpositivist, constructivist / interpretivist) is also recommended; rationale. The rationale should briefly discuss the justification for choosing that theory, approach, method or technique rather than other options available; the assumptions and limitations implicit in those choices and how those choices influence study conclusions and transferability.	10

For peer review only

		As appropriate the rationale for several items might be discussed together.	
Researcher characteristics and reflexivity	#6	Researchers' characteristics that may influence the research, including personal attributes, qualifications / experience, relationship with participants, assumptions and / or presuppositions; potential or actual interaction between researchers' characteristics and the research questions, approach, methods, results and / or transferability	10
Context	#7	Setting / site and salient contextual factors; rationale	7
Sampling strategy	#8	How and why research participants, documents, or events were selected; criteria for deciding when no further sampling was necessary (e.g. sampling saturation); rationale	7-9
Ethical issues pertaining to human subjects	#9	Documentation of approval by an appropriate ethics review board and participant consent, or explanation for lack thereof; other confidentiality and data security issues	9
Data collection methods	#10	Types of data collected; details of data collection procedures including (as appropriate) start and stop dates of data collection and analysis, iterative process, triangulation of sources / methods, and modification of procedures in response to evolving study findings; rationale	8-10

For peer review only

Data collection instruments and technologies	#11	Description of instruments (e.g. interview guides, questionnaires) and devices (e.g. audio recorders) used for data collection; if / how the instruments(s) changed over the course of the study	9-10
Units of study	#12	Number and relevant characteristics of participants, documents, or events included in the study; level of participation (could be reported in results)	11
Data processing	#13	Methods for processing data prior to and during analysis, including transcription, data entry, data management and security, verification of data integrity, data coding, and anonymisation / deidentification of excerpts	10
Data analysis	#14	Process by which inferences, themes, etc. were identified and developed, including the researchers involved in data analysis; usually references a specific paradigm or approach; rationale	10
Techniques to enhance trustworthiness	#15	Techniques to enhance trustworthiness and credibility of data analysis (e.g. member checking, audit trail, triangulation); rationale	10
**Results/findings**			
Syntheses and interpretation	#16	Main findings (e.g. interpretations, inferences, and themes); might include development of a theory or model, or integration with prior research or theory	11-12

For peer review only

Links to empirical data	#17	Evidence (e.g. quotes, field notes, text excerpts, photographs) to substantiate analytic findings	11-17
**Discussion**			
Intergration with prior work, implications, transferability and contribution(s) to the field	#18	Short summary of main findings; explanation of how findings and conclusions connect to, support, elaborate on, or challenge conclusions of earlier scholarship; discussion of scope of application / generalizability; identification of unique contributions(s) to scholarship in a discipline or field	18-20
Limitations	#19	Trustworthiness and limitations of findings	18-19
**Other**			
Conflicts of interest	#20	Potential sources of influence of perceived influence on study conduct and conclusions; how these were managed	26
Funding	#21	Sources of funding and other support; role of funders in data collection, interpretation and reporting	26

The SRQR checklist is distributed with permission of Wolters Kluwer © 2014 by the Association of American Medical Colleges. This checklist was completed on 06. June 2019 using https://www.goodreports.org/, a tool made by the EQUATOR Network in collaboration with Penelope.ai

For peer review only
